# Recent Trends in the Development of Green Analytical Sample Preparation Methods Using Advanced Materials

**DOI:** 10.1002/jssc.70177

**Published:** 2025-05-26

**Authors:** Eduardo Carasek, Amanda Vitória Santos, Francielle Crocetta Turazzi, Lucas Morés, Luciane Effting, Guilherme Mariz de Oliveira Barra

**Affiliations:** ^1^ Departamento de Química Universidade Federal de Santa Catarina Florianópolis Brazil; ^2^ Departamento de Engenharia Mecânica Universidade Federal de Santa Catarina Florianópolis Brazil

**Keywords:** Analytical Greenness Metric for Sample Preparation, chromatographic separation, conductive polymers, metal–organic frameworks, molecularly imprinted polymers

## Abstract

Recent concern about the impact of environmental preservation and the health of living beings has opened new avenues for scientific research. In this context, contemporary analytical chemistry has been marked by the development of green analytical methodologies, which aim to reduce the use of toxic reagents and minimize the environmental impact of analytical processes. Progress in this area involves the optimization of sample preparation techniques and the use of new functional materials, which contribute to a more sustainable and efficient analysis. Among these methodologies, miniaturized sample preparation techniques stand out, as they use smaller volumes of solvents and offer high sensitivity and selectivity. The use of advanced materials, such as molecularly imprinted polymers, MOFs, and conductive polymers, has driven innovation in analytical procedures regarding complex matrices, including environmental, food, and biological samples. These materials offer high selectivity and stability, improving efficiency in the extraction and detection of specific analytes. This review explores the integration of sustainable and green methodologies. It critically highlights applications and evaluates them using the Analytical Greenness Metric for Sample Preparation, based on publications from the past 6 years.

**Abbreviations**
AFM: atomic force microscopy
AGREE: Analytical Greenness
AGREEprep: Analytical Greenness Metric for Sample Preparation
APGC–MS/MS: gas chromatography with atmospheric pressure chemical ionization‐tandem mass spectrometry
CMPs: conjugated microporous polymers
CPs: conductive polymers
DμSPE: dispersive micro‐solid‐phase extraction
DESs: deep eutectic solvents
DLLME: dispersive liquid–liquid microextraction
DLSE: dispersive liquid solid‐phase extraction
DMMIPs: dual‐template magnetic molecularly imprinted polymers
DPX: disposable pipette extraction
DSPE: dispersive solid‐phase extraction
EC‐SPME: electrochemically controlled‐solid‐phase microextraction
GAC: Green Analytical Chemistry
GAPI: Green Analytical Procedure Index
GC/FPD: gas chromatography/flame photometric detector
GC‐ECD: gas chromatography‐electron capture detector
GC‐FID: gas chromatography‐flame Ionization detector
HPLC–DAD: high‐performance liquid chromatography–diode array detector
HPLC‐FLD: high‐performance liquid chromatography‐fluorescence detector
ILs: ionic liquids
IS‐SPE: in‐syringe‐solid‐phase extraction
LLE: liquid–liquid extraction
LLSMME: liquid–liquid–solid membrane microextraction
LPME: liquid‐phase microextraction
MDSPE: magnetic dispersive solid‐phase extraction
MEPS: microextraction by packed sorbent
MIP: molecularly imprinted polymer
MISPE: molecularly imprinted solid‐phase extraction
MMIPs: magnetic molecularly imprinted polymers
M‐SA‐DSPE: modified magnetic‐based solvent‐assisted dispersive solid‐phase extraction
MSPE: magnetic solid‐phase extraction
NIP: non‐imprinted polymer
PAHs: polycyclic aromatic hydrocarbons
PCMs: porous carbon materials
QuEChERS: quick, easy, cheap, effective, rugged, and safe
SBSE: stir‐bar sorptive extraction
SC‐μSPE: spin column micro‐solid‐phase extraction
SHS: switchable hydrophilicity solvents
SPMS: Sample Preparation Metric of Sustainability
SUPRAS: supramolecular solvents
TF‐SPME: thin film‐solid‐phase microextraction
UA‐d‐SPE: ultrasonic assisted‐dispersive‐solid‐phase extraction
UHPLC–DAD: ultra‐high performance liquid chromatography–diode array detector
UPLC‐DAD: ultra‐performance liquid chromatography‐diode array detector
UPLC‐HRMS: ultra‐high‐performance liquid chromatography‐high‐resolution mass spectrometry
UPLC–MS/MS: ultra‐performance liquid chromatography‐tandem mass spectrometry
WAC: white analytical chemistry
ZIF: zeolitic imidazole framework

## 1 Introduction

As a fundamental stage in analytical procedures, sample preparation significantly impacts the accuracy and sensitivity of the results [1]. This stage is one of the main ones within an analytical system, seeking to resolve a problem through processes and tools that result in truthful responses. This step involves pre‐concentration, purification, and the extraction of target analytes from several complex matrices, ensuring reliable analytical outcomes [2, 3, 4, 5]. Various methodologies have been developed to optimize these processes, improving efficiency and analytical performance. Classical techniques, such as liquid–liquid extraction (LLE) and SPE, have long been employed for environmental, food, and biological sample analysis. However, these techniques present challenges related to time consumption, operator safety, and excessive waste generation [6].

Growing environmental and health concerns have driven the development of miniaturized sample preparation techniques [2]. These methods, which typically require minimal volumes of extraction phases relative to the sample, offer a sustainable alternative to traditional approaches [7]. The introduction of solid‐phase microextraction (SPME) by Pawliszyn in 1990 marked a pivotal advancement in microextraction techniques, such as stir‐bar sorptive extraction (SBSE), dispersive liquid–liquid microextraction (DLLME), and thin‐film SPME (TF‐SPME), among other variations [4, 5, 8]. Refinements in these techniques have addressed initial limitations and have enhanced extraction speed, capacity, and stability while improving surface area flexibility, film thickness, robustness, and coating durability [7]. Additionally, the automation of these techniques has further improved accuracy, precision, and environmental sustainability compared to manual procedures [9].

Beyond selecting an appropriate extraction technique, the choice of the extraction material plays a critical role in the efficiency of sample preparation. Although commercial materials are widely used, their high costs, limited selectivity, and challenges in extracting polar analytes have driven the search for alternative materials [10, 11].

Consequently, significant efforts have been directed toward developing new extraction materials that enhance selectivity, sensitivity, and cost‐effectiveness [12]. These novel materials include deep eutectic solvents (DESs), molecularly imprinted polymers (MIPs), ionic liquids (ILs), conjugated microporous polymers (CMPs), supramolecular solvents (SUPRAS), switchable hydrophilicity solvents (SHS), porous carbon materials (PCMs), COFs, metal–organic frameworks (MOFs), conductive polymers (CPs), and natural polymers such as cellulose and chitin/chitosan [2, 5, 6, 13, 14].

Among these materials, MIPs, MOFs, and CPs were selected on the basis of their distinctive properties and advantages. MIPs exhibit high selectivity and specificity, enabling efficient extraction of target analytes from complex matrices. Their chemical and thermal stability further supports their use in diverse sample preparation techniques. MOFs, characterized by high surface area, tunable porosity, and selective adsorption capacity, are promising for compound extraction and pre‐concentration. CPs, with their versatility, electrostatic interaction capabilities, and affinity for various compound classes, expand the scope of sample preparation applications. These materials were chosen due to their demonstrated effectiveness, robustness, and practical applicability, as supported by extensive literature [2, 15–17].

Regarding sustainability focused on these materials, the shift toward sustainable analytical chemistry has led to the adaptation of sample preparation methods to reduce environmental impact, including these materials as an alternative to improve this environment‐friendly condition. Several assessment tools have been developed to evaluate the environmental friendliness of analytical techniques, facilitating the comparison of sustainability across methods [18]. Examples include white analytical chemistry (WAC) [19], eco‐scale [20], Green Analytical Procedure Index (GAPI) [21], Analytical Greenness (AGREE) [22], Sample Preparation Metric of Sustainability (SPMS) [23], and the AGREE Metric for Sample Preparation (AGREEprep) [24]. Because each tool applies distinct criteria, sustainability comparisons should use the same metric to ensure consistency. Although GAPI assesses the entire analytical workflow, AGREEprep focuses exclusively on the sample preparation. AGREEprep is particularly valuable due to its scoring system and visual representation, making it easier to identify environmentally favorable aspects of the method [25].

This review presents a comprehensive examination of methodologies integrating sustainable and green chemistry principles, emphasizing the role of MIPs, MOFs, and CPs in modern sample preparation. It synthesizes research from the past 6 years and demonstrates the effectiveness of these advanced materials in green analytical techniques through identification by chromatographic instrumentation. Additionally, the study evaluates their environmental performance using the AGREEprep software, providing valuable insights into the sustainability of these methods.

## 2 Assessment of Sustainable Parameters Using AGREEprep

Green Analytical Chemistry (GAC) is a growing field that aims to develop more sustainable analytical methodologies, reducing the environmental impact of laboratory practices. In‐line with the principles of green chemistry, GAC applies these concepts to all analytical stages, including sample collection, preparation, and analysis, striving for efficient and environmentally responsible methods in various areas, such as the environment, health, and food safety [19, 25].

In the context of GAC, several tools have been developed to assess and quantify the sustainability of analytical methods, enabling scientists to identify and adopt more environmentally friendly practices. Among these tools, AGREEprep stands out as it provides a quantitative assessment of the environmental impact of analytical methods and sample preparation steps, based on several criteria [24].

Among the criteria defined in the metric, this review establishes specifications to evaluate each of the mentioned applications equitably for each parameter [24]. The following is the list of the 10 criteria.

### 2.1 Criterion 1. Favor In Situ Sample Preparation

Considering that sample preparation represents the most labor‐intensive step of the analytical procedure, reducing time, energy consumption, and material usage becomes a relevant consideration. The first AGREEprep criterion (weight 1) encourages in situ sample preparation, integrating sampling with sample preparation to reduce processing time and preserve sample integrity by eliminating the need for storage and transport. In‐line/in situ sampling, performed directly at the location of the sample, receives the maximum score (1.0). On‐line/in situ sampling (score: 0.66) involves sample preparation at the collection site using permanently installed devices. In this process, both sampling and preparation are performed simultaneously, with operation often automated. A score of 0.33 (on site) is given when the preparation device is transported to the sampling site. Finally, the minimum score (0.0) is given when sample preparation is ex situ and occurs in the laboratory, after sample collection and transportation [24].

### 2.2 Criterion 2. Use Safer Solvents and Reagents

The second criterion, with a high weight (weight 5), promotes the use of safer and environmentally friendly materials. The maximum score is achieved when sample preparation does not involve toxic substances. The minimum score (0.0) is assigned when the use of hazardous materials exceeds 10 mL or 10 g [24].

### 2.3 Criterion 3. Target Sustainable, Reusable, and Renewable Materials

The characteristics of materials used should be evaluated to minimize their environmental impact, which justifies assigning weight 2 to this criterion. Sustainable materials are defined by their low environmental impact throughout their life cycle. Renewable materials are derived from natural sources that can be replenished, reducing reliance on finite resources. The use of reusable materials aims to reduce waste generation during sample preparation, thereby decreasing environmental impact. The use of biologically sourced materials yields a higher score compared to fossil‐based chemicals and other non‐renewable substances. The maximum score (1.0) is assigned when renewable and sustainable materials are reused. A score of 0.75 is given when more than 75% of the materials and reagents used are sustainable or renewable. The score of 0.5 is assigned in two cases: Either when the materials are reused but are neither renewable nor sustainable, or when 50%–75% of the materials are sustainable or renewable but can only be used once. In cases where 25%–50% of the reagents are sustainable or renewable, the assigned score is 0.25. The minimum score (0.0) is assigned to non‐reusable materials or when less than 25% of the materials are reusable or sustainable [24].

### 2.4 Criterion 4. Minimize Waste

Waste disposal and treatment generated during sample preparation are critical due to the properties of the substances involved. The AGREEprep addresses this concern in Criterion 4 (weight 4), favoring procedures that minimize waste generation during the preparation stage over those that produce larger amounts of waste. In sample preparation, the material inputs are treated as waste because the materials used are not incorporated into the final product (analytical result). This criterion exclusively evaluates the quantity of waste generated, as the associated risks are addressed in other criteria [24].

### 2.5 Criterion 5. Minimize Amounts of Sample, Chemicals, and Materials

The sample size used directly impacts time and energy consumption, as well as waste generation. The reduction of the sample volume should be carried out in a manner that preserves its representativeness, ensuring that the analyzed fraction retains the characteristics and composition of the original material. This criterion (weight 2) encourages the miniaturization of sample preparation, facilitating process portability and automation [24].

### 2.6 Criterion 6. Minimize Sample Throughput

Criterion 6 (weight 3) promotes methods that result in high analytical throughput, which can be achieved by rapid methods or parallel sample preparation using systems like 96‐ or 24‐well plates. Pena‐Pereira et al. report the analytical throughput benefits of preparing samples in parallel, as shown in Table 1 [26].

**TABLE 1:** Sample throughput calculations.

Sample preparation technique	Single sample preparation time	Samples prepared in parallel	Sample throughput per hour
Soxhlet extraction	24 h	1	0.042
Soxhlet extraction	24 h	6	0.25
DLLME	15 min	1	4
DLLME	15 min	8	32
SPME	60 min	1	1
SPME	60 min	96	96

Abbreviations: DLLME, dispersive liquid–liquid microextraction; SPME, solid‐phase microextraction.

*Source*: Adapted from Pena‐Pereira et al. [26].

### 2.7 Criterion 7. Integrate Steps and Promote Automation

Sample preparation often involves lengthy and laborious steps, increasing energy consumption, contamination risk, and material loss. To address these challenges, AGREEprep promotes the integration and automation of these steps, aiming to reduce sample and energy usage, minimize waste generation, and limit human intervention. This approach also enhances analytical efficiency and frequency, making sample preparation more sustainable and rapid. This criterion is evaluated with a weight of 2 and is calculated as the product of the sub‐scores assigned to the number of procedural steps and the degree of automation in the sample preparation process. The scoring for the number of steps is assigned as follows: Procedures with up to two steps receive the maximum score (1.0), whereas those with three, four, and five steps are scored at 0.75, 0.5, and 0.25, respectively. Processes involving six or more steps receive a score of zero (0.0). The degree of automation is scored according to the level of system automation: Fully automated systems receive the maximum score (1.0), semi‐automated systems are scored at 0.5, and manual systems receive the lowest score (0.25) [24].

### 2.8 Criterion 8. Minimize Energy Consumption

This criterion is weighted 4. Energy consumption throughout the sample preparation process should be assessed to enhance process greening, with efforts to minimize it whenever possible. This reduction not only minimizes environmental impacts but also contributes to the laboratory's economic efficiency. By evaluating each stage in the process, energy‐efficient techniques can be identified and adopted. The simultaneous preparation of multiple samples reduces energy consumption when using the same device, as its energy expenditure is distributed across the number of samples processed [24].

### 2.9 Criterion 9. Choose the Greenest Possible Post‐Sample Preparation Configuration for Analysis

The final stage of sample preparation is critical, as its choice depends on analytical requirements and instrument availability. Criterion 9 (weight 2) is responsible for assessing the impact of the final stage on sample preparation. Green sample preparation principles suggest selecting simple instrumentation that requires low energy and chemical input. For this review, only studies that employed chromatographic instrumentation will be reported. When a gas chromatography (GC) with non‐MS detection is used, a score of 0.5 is assigned. In the case of analyses conducted with a GC–MS, the score is reduced to 0.25. A score of 0.25 is also assigned to LC due to the consumption of mobile phases, which are commonly composed of organic solvents [24].

### 2.10 Criterion 10. Ensure Safe Procedures for the Operator

Operator protection is essential during sample preparation, and selecting materials that ensure this safety should be carefully considered when developing the method. Criterion 10 (weight 3) scoring is based on reducing the use of substances that are toxic to life and the environment and that present bioaccumulation potential and product flammability, thereby promoting safer and more sustainable laboratory practices. The score for this criterion is assigned based on the number of chemical, physical, or biological risks to which the operator is exposed. When no risks are identified, the assigned score is 1.0. If one risk is identified, the score is 0.75; for two risks, the score is 0.5; for three risks, the score is 0.25; and when four or more risks are identified, the assigned score is 0.0 [24].

## 3 Materials in Green Sample Preparation Methodologies

### 3.1 Molecularly Imprinted Polymers

#### 3.1.1 Fundamentals

MIPs are polymers specifically designed to recognize a target molecule or a group of structurally similar compounds with significant selectivity. This characteristic is attributed to binding sites within the polymer, which possess size, shape, and binding affinity that are conferred by the analyte of interest itself. The mechanism is often compared to a “lock and key” model [27].

The first use of MIP was reported in 1972 by Wullf and Sarhan, describing the synthesis of a polymer with selective sites for enantiomeric separation of sugar racemates. However, it was only from 1993 onwards that a significant number of publications on the use of this material appeared in the scientific literature [28, 29].

In the synthesis of MIPs, interactions are established between the molecule of interest (template) and functional monomers in the presence of a crosslinking agent and a porogenic solvent. After polymerization, a molecular template is extracted, and binding sites are created. A dummy model can be used to reduce the effect of overestimation during analyte extraction. Typically, the dummy model has a chemical structure similar to that of the analyte. The resulting MIP is chemically stable, robust, and resistant to a wide range of pH, solvents, and temperatures [30]. The molecular recognition ability of the MIP is often evaluated by comparison with a non‐imprinted polymer (NIP). This material is synthesized under the same conditions as MIP, except for the addition of the template, functioning as a control polymer [31]. The comparison between MIP and NIP is essential for verifying selectivity and allows the determination of whether the recognition of the target molecule is actually due to molecular imprinting or just nonspecific adsorption, adsorption efficiency, optimization of the synthesis process, and evaluation of stability and reusability. The comparison can be made with adsorption isotherms, partition coefficient (Kd), and imprinting factor (IF) [32].

In recent years, the production of MIPs has improved; a wide range of studies have been published in the literature, and information can be found about the material, its application, synthesis methods, and selection of reagents. The application of the synthesized material is an important factor to be considered, and the choice of appropriate reagents is essential to obtain a material with the desired properties [15, 31–33]. Moreover, MIPs are frequently combined with other materials, such as magnetic materials (Fe_3_O_4_) [34, 35], silica (SiO_2_) [36], graphene oxide (GO) [37], multi‐walled carbon nanotubes (MWCNTs) [38], gold nanoparticles [39], and others, aggregating the properties of both materials.

In chromatography applications, MIPs are commonly employed for pre‐concentration or cleanup of organic compounds from various matrices, including aqueous samples [40], foods [34, 41], beverages [35, 42], urine [43], plasma [44], and others. Furthermore, MIPs can be exploited as an electrochemical sensor [38, 45] or as a selective sorbent for ions (ion‐imprinted polymer—IIP) [46].

A brief description of some applications of MIPs in sample preparation methods published in the literature is presented in the next section, with critical reviews in different samples.

#### 3.1.2 Recent Applications of MIPs in Sample Preparation

##### 3.1.2.1 MIPs in Environmental Matrices

For environmental samples, the major uses of MIPs are in water samples, like rivers, lakes, or tap water. One study reported research in which they were employed with another type of sample, namely, wet‐blue and crust leather [47].

Lu et al. [47] demonstrated a successful strategy for the synthesis of magnetic multi‐template MIPs (M‐mt‐MIPs) for the adsorption of chlorophenols (2‐chlorophenol, 4‐chlorophenol, 2,6‐dichlorophenol, 2,4‐dichlorophenol, 4‐nitrophenol, and 4‐*tert*‐amylphenol) onto wet‐blue and crust leather samples using magnetic solid‐phase extraction (MSPE). To explore the adsorption behavior of M‐mt‐MIPs for target chlorophenols in aqueous solution, static adsorption, dynamic adsorption, selectivity adsorption, and reuse were studied. The analytical performance of the method and spiked recovery experiment with real leather samples were also investigated; LOQ values ranging from 1.07 to 1.62 µg L^−1^ and enrichment factors from 35.2 to 108 times were obtained, which demonstrates the high sensitivity of the method. The material was applied in the analysis of chlorophenols in tannery wastewater, wet‐blue, and crust leather, and no chlorophenol was detected in these three samples, although satisfactory recoveries were obtained, in the range of 73.95%–109.7%. The green aspects of the method were evaluated, and a score of 0.33 was obtained. The sample preparation ex situ (Criterion 1), use of hazardous materials (Criterion 2), waste generation (Criterion 4), at least one sample prepared in an hour (Criterion 6), and many steps without automation are not favorable in analysis and decrease a score for the method.

Baeza et al. [48] reported the development of a highly sensitive online molecularly imprinted solid‐phase extraction (MISPE) methodology for the determination of fluoroquinolones, enrofloxacin, norfloxacin, lomefloxacin, enoxacin, levoxacin, ciprofloxacin, sarafloxacin, and danofloxacin in water. The MISPE method is an online pre‐concentration SPE, and in this method, the entire sample is transferred to the chromatographic system, generating improved LODs. The method obtained LOQs in the range of 0.4 to 2.2 ng L^−1^ and recoveries of 66%–101%. The analysis of Quibú River water was carried out at four different points of its flow, finding residues of norfloxacin, enrofloxacin, and danofloxacin. According to the authors, this fact makes sense, because norfloxacin is an antibiotic that is more widely used in a range of treatments than others that belong to this family. The green aspects of the method were analyzed, and the score of 0.26 was obtained. Only two criteria were considered green, Criteria 1 and 10, namely, sample preparation placement online and the issue of not being hazardous for operators. As unfavorable characteristics, the method uses a large sample quantity and, consequently, produces a high quantity of residue, and less than one sample is performed per hour.

**FIGURE 1:**
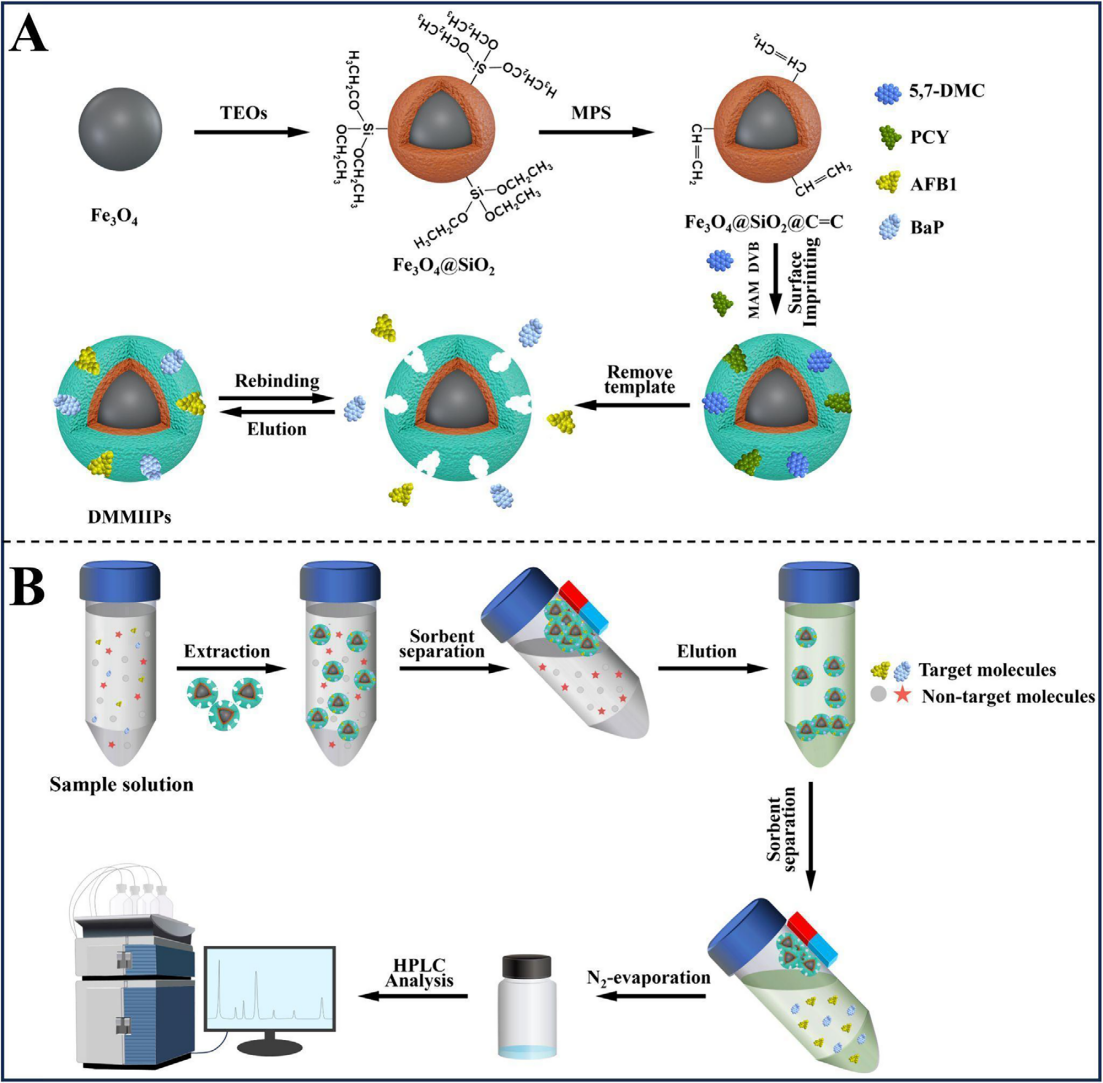
Schematic diagrams of (A) dual‐template magnetic molecularly imprinted polymer sorbent fabrication and (B) the proposed magnetic solid‐phase extraction‐HPLC procedure for aflatoxin B1 and benzo(α)pyrene. 5,7‐DMC, 5,7‐dimethoxycoumarin. *Source*: Reprinted from Wang et al. [49] with permission from Elsevier.

Wang et al. [49] prepared dual‐template magnetic MIPs (DMMIPs) using surface molecularly imprinted technology to simultaneously recognize and extract aflatoxin B1 and benzo(α)pyrene. Dummy templates are compounds or structural fragments similar to target templates using 5,7‐dimethoxycoumarin (5,7‐DMC) and pyrene (PYR) as dual dummy templates. The LODs and LOQs were 0.134 and 0.402 µg L^−1^ for aflatoxin B1 and 0.107 and 0.351 µg L^−1^ for benzo(α)pyrene, and recoveries in water ranged from 89.0% to 110.3% for aflatoxin B1 benzo(α)pyrene, respectively. The score of the method calculated by AGREEprep was 0.38, with good aspects for Criteria 8 and 10, namely, the low consumption of energy and not being hazardous for operators. But the negative aspects included sample preparation ex situ, a large amount of hazardous material, and generation of residues. The material synthesis steps and MSPE for sample preparation are shown in Figure 1.

Other applications of MIPs for environmental matrices, the sample preparation techniques, and chromatographic instrumentation are presented in Table 2 [37, 40, 47–57].

**TABLE 2:** Recent applications of molecularly imprinted polymers in sample preparation methods applying chromatographic separation in environmental matrices.

Material	Sample preparation technique	Analytes	Sample	Instrument	Linear range	LOQ	Recovery (%)	AGREEprep	Reference
MIPs/GO	SPME	Triphenyl phosphate	River and lake water	GC/FPD	0.0007–124 ng mL^−1^	0.0004 ng mL^−1^	70–110	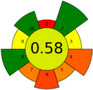	[37]
MMIPs	MSPE	Bisphenol A	River and lake water	HPLC‐UV	2.0–1000 µg L^−1^	1.6 µg L^−1^	86.26–94.43	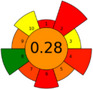	[40]
M‐mt‐MIPs	MSPE	2‐Chlorophenol, 4‐chlorophenol, 2,6‐dichlorophenol, 2,4‐dichlorophenol, 4‐nitrophenol, and 4‐*tert*‐amylphenol	Tannery wastewater, wet blue, and crust leather	HPLC–DAD	2–200 µg L^−1^	1.07–1.62 µg L^−1^	73.95–109.7	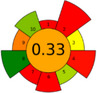	[47]
MIP	MISPE	Enrofloxacin, norfloxacin, lomefloxacin, enoxacin, levoxacin, ciprofloxacin, sarafloxacin, and danofloxacin	Surface water	HPLC‐FLD	0.7–666 µg L^−1^	0.4–2.2 µg L^−1^	66–101	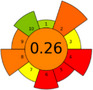	[48]
DMMIPs	MSPE	Aflatoxin B1 and benzo(α)pyrene	Lake and tap water	HPLC	0.5–50 µg L^−1^	0.351–0.402 µg L^−1^	89.0–110.3	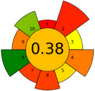	[49]
MIP	MISPE	Phenol, alkylphenols, and chlorophenols	Spiked seawater and produced water	UHPLC–DAD	0.5–3000 µg L^−1^	—	81–107	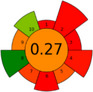	[50]
MIP	SPE	Chlorpyrifos and diazinon	Water	HPLC‐UV	50–1000 µg L^−1^	0.23–0.41 µg L^−1^	79–104	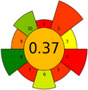	[51]
MMIP	DSPE	PAHs	River water and produced water	APGC–MS/MS	2–50 000 pg mL^−1^	2–200 pg mL^−1^	4.5–97	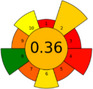	[52]
MIP	MISPE	Bisphenol A	River water	HPLC–DAD	0.1–1 ng µL^−1^	0.045 ng µL^−1^	85.4–96.1	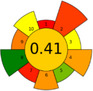	[53]
MIP	MISPE	Imazapyr, imazapic, and imazethapyr	Surface water	HPLC–DAD	0.15–200.0 µg L^−1^	0.15–0.29 µg L^−1^	92–102	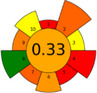	[54]
MIP	SBSE	Paraquat	Tap and river water	HPLC	100–10 000 ng L^−1^	27 ng L^−1^	70.0–96.1	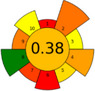	[55]
MIP	MSPE	Norfloxacin	Lake water	UHPLC‐UV	0.05–300 µg mL^−1^	—	90.9–97.1	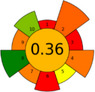	[56]
dt‐MIPs	DSPE	Norfloxacin and enrofloxacin	Lake, sea, and tap water	HPLC	1–200 µg L^−1^	0.67–0.98 µg L^−1^	80.9–101.0	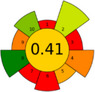	[57]

Abbreviations: APGC–MS/MS, GC with an atmospheric pressure chemical ionization‐tandem mass spectrometry; DSPE, dispersive solid‐phase extraction; GC/FPD, gas chromatography/flame photometric detector; HPLC–DAD, high‐performance liquid chromatography–diode array detector; MIPs/GO, molecularly imprinted polymer/graphene oxide; MISPE, molecularly imprinted solid‐phase extraction; M‐mt‐MIPs, magnetic multi‐template molecularly imprinted polymers; MSPE, magnetic solid‐phase extraction; UHPLC–DAD, ultra‐high performance liquid chromatography–diode array detector.

In AGREEprep analysis, the values obtained ranged from 0.26 to 0.58. This range suggests varying levels of “greenness” among the methods evaluated. The relatively low upper limit implies that none of the methods analyzed were highly sustainable or environmentally friendly, and the lower limit (0.26) indicates that some methods were particularly harmful to the environment. Most of the work achieved metrics between 0.3 and 0.4 due to the generation of waste, the amount of sample that was used, and the use of materials considered hazardous, and the fact that most methods scored in this range indicates a direction in the environmental impact of the techniques analyzed.

##### 3.1.2.2 MIPs in Food Matrices

The application of MIPs in food samples is widespread for endogenous compounds, such as Rhodamine B from chili powder samples [58] and cholesterol in milk samples [26], or contaminants like mycotoxins in maize samples [59], and pesticides in vegetables [55], honey [60], and juice [34]. The main uses of MIPs are based on a single analyte, due to the specificity of site recognition, but some studies are found for a whole class of analytes.

Wang et al. [61] explored the synthesis of the MIP with a single functional and cross‐linking monomer to simplify the preparation and application for a class of estrogens, estrone, 17β‐estradiol, estriol, ethinyl estradiol, and estradiol benzoate. For this purpose, 2,5‐divinylterephthalaldehyde was used, together with estradiol as a template, a solvent, and a radical initiator, and this reduced the number of variables encountered in the traditional synthesis. The MIP obtained was employed in SPME. The ranges for the LOQs are 0.26–0.87 ng kg^−1^, and the MIP‐coated fiber can be reused more than 60 times without any marked reduction in the enrichment efficiency. All samples presented estrogens in non‐spiked milk samples. The analysis by AGREEprep showed a score of 0.35 due to sample preparation placement ex situ, large amounts of hazardous, non‐renewable materials that generate a large amount of waste, in addition to few samples made per hour, many steps and without any automation, and liquid chromatography equipment used for quantification. In this case, the parameters of the method are not favorable in a green evaluation.

In another study, Dinali et al. [42] described the synthesis of a mesoporous MIP on the surface of silica nanoparticles (core@mMIP) to be applied as the adsorbent in microextraction by packed sorbent (MEPS) for selective determination of pesticides (pyriproxyfen, deltamethrin, and etofenprox) in apple juice. For both analytes, the LOQs obtained were 0.02 µg mL^−1^. The results obtained from the fresh juice sample showed that residues of pyriproxyfen and deltamethrin were found below the LOQ of the method. For processed whole apple juice, the presence of etofenprox also was detected below the LOQ of the method, but the deltamethrin residue was quantified. A score of 0.64 was obtained for the method in AGREEprep. The favorable criteria are 5 and 6, relating to the size economy of the sample and sample throughput, respectively, emphasizing that the smaller sample and the number of samples per hour are important for method development. But the negative aspects were Criteria 1, 3, 7, and 9, relative to sample preparation placement in the laboratory, the use of non‐renewable reagents, materials that are used only once, five steps of sample preparation in manual systems, and the use of liquid chromatography as the quantification equipment.

Abdulhussein et al. [60] synthesized new and specific sorbent dual‐template MIPs (dt‐MIPs) for extracting two neonicotinoids, thiamethoxam and thiacloprid, with active sides to be used in the removal and clean‐up of target components from light and dark honey samples. The recoveries obtained for thiamethoxam and thiacloprid in light honey and dark honey samples were 96.8%–106.5% and 95.3%–104.4%, respectively, and in comparison to the standard approach of the QuEChERS (Quick, Easy, Cheap, Effective, Rugged, and Safe) method, recoveries of 93.0%–99.6% for thiamethoxam and 92.7%–98.8% for thiacloprid in light and dark honey samples were obtained. The score by AGREEprep for the method developed is 0.37. Some aspects explain the value, such as sample preparation placement ex situ (Criterion 1), many hazardous materials (Criterion 2), six steps of sample preparation and no automation of systems (Criterion 7), and the use of liquid chromatography as quantification equipment (Criterion 9). The positive criteria are the size economy of the sample (Criterion 5), low energy consumption per analysis (Criterion 8), and low hazard to operator's safety (Criterion 10).

Table 3 shows more applications of MIPs for food samples with chromatographic quantification [34, 35, 40–42, 49, 54, 55, 58–70].

**TABLE 3:** Recent applications of molecularly imprinted polymers in sample preparation methods applying chromatographic separation in food matrices.

Material	Sample preparation technique	Analytes	Sample	Instrument	Linear range	LOQ	Recovery (%)	AGREEprep	Reference
Fe_3_O_4_@SiO_2_‐MDMIP	SS‐MSPME	*p*‐Coumaric acid and ferulic acid	Pomegranate, grape, and orange	HPLC‐UV	0.5–300 ng mL^−1^	0.15–0.3 ng mL^−1^	85.12–94.96	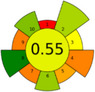	[34]
Fe_3_O_4_/SiO_2_/MIP	MDSPE	Cholesterol	Milk	HPLC–DAD	38.5.0–25 000 µg L^−1^	38.5 µg L^−1^	92–97	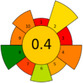	[35]
MMIP	MSPE	Bisphenol A	Milk powder	HPLC‐UV	4.0–1000 µg L^−1^	8.3 µg L^−1^	86.26–94.43	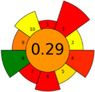	[40]
MIP	MSPE	Zearalenone	Wheat	HPLC–DAD	1.56–12.5 ng g^−1^	1.56 ng g^−1^	92.1–96.0	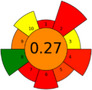	[41]
core@mMIP	MEPS	Pyriproxyfen, deltamethrin, and etofenprox	Whole processed apple juice	HPLC	0.02–10 µg mL^−1^	0.02 µg mL^−1^	80–100	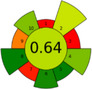	[42]
DMMIP	MSPE	Aflatoxin B1 and benzo(α)pyrene	Peanut oil, soybean oil, corn oil, and rapeseed oil	HPLC	0.5–50 µg L^−1^	0.351–0.402 µg L^−1^	86.2–113.2	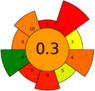	[49]
MIP	MISPE	Imazapyr, imazapic, and imazethapyr	Rice	HPLC–DAD	0.15–200.0 µg L^−1^	0.15–0.29 µg L^−1^	86–107	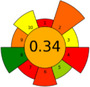	[54]
MIP	SBSE	Paraquat	Lettuce	HPLC	0.02–0.85 mg kg^−1^	0.017 mg kg^−1^	85.1–88.4	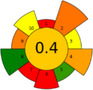	[55]
MIP	PT‐SPE	Rhodamine B	Chili powder	HPLC‐UV/Vis	0.005–15 mg kg^−1^	0.0015 mg kg^−1^	85.4–102.0	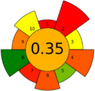	[58]
SPMIP	μ‐SPE	Zearalenone	Maize	HPLC‐FLD	10–200 ng mL^−1^	8 ng mL^−1^	74.95–88.41	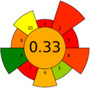	[59]
MIP	MSPE	Thiamethoxam and thiacloprid	Honey	UHPLC–MS/MS	1–100 µg L^−1^	2.1–5 µg L^−1^	80.0–104.0	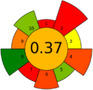	[60]
MIP	SPME	Estrone, 17β‐estradiol, estriol, ethinylestradiol, and estradiol benzoate	Milk	UHPLC–MS/MS	0.5–10 000 ng kg^−1^	0.26–0.87 ng kg^−1^	84.3–105	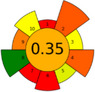	[61]
MIP	SBSE	Thiabendazole and carbendazim	Orange peel	HPLC	25–1500 µg L^−1^	0.27–0.30 µg L^−1^	—	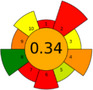	[62]
Fe_3_O_4_/SiO_2_/MIP	MDSPE	Chlortetracycline, doxycycline, oxytetracycline, and tetracycline	Milk	HPLC–DAD	10.0–100.0 µg L^−1^	0.26–0.60 µg L^−1^	96–102	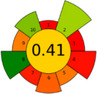	[63]
MMIP	MDSPE	Rifaximin	Milk	HPLC‐UV	0.05–10 mg L^−1^	0.03 mg L^−1^	86.67–99.47	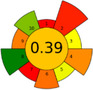	[64]
MMIP	MDSPE	Captan	Skim and pasteurized milk	HPLC	0.01–100 ppb	—	72.8–83.97	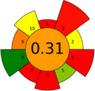	[65]
MIP	SPE	Citrinin	Red yeast rice	HPLC	25–4000 µg L^−1^	25 µg L^−1^	76–91	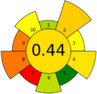	[66]
MIP	SPME	Ochratoxin A	Beer	HPLC‐FLD	0.1–10.0 ng mL^−1^	0.2 ng mL^−1^	81.8–82.8	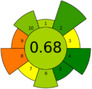	[67]
MIP	MISPE	Catechin	Red angico, Jabuticaba, and Umbu	HPLC‐UV	45–700 mg L^−1^	40 mg L^−1^	108.5–101.9	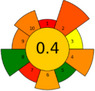	[68]
MIP	MISPE	Lincomycin	Milk	HPLC‐UV	0.08–2 µg mL^−1^	0.08 µg mL^−1^	80–89	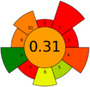	[69]
MYS‐MMIPs	MSPE	17β‐estradiol	Milk powder	HPLC	1–300 µg kg^−1^	—	88.3–102.4	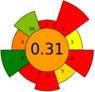	[70]

Abbreviations: μ‐SPE, dispersed solid‐phase extraction; Fe_3_O_4_/SiO_2_/MIP, magnetic molecularly imprinted polymer; Fe_3_O_4_@SiO_2_‐MDMIP, magnetic dual‐template molecularly imprinted polymer; HPLC, high‐performance liquid chromatography; MDSPE, magnetic dispersive solid‐phase extraction; MISPE, molecularly imprinted solid‐phase extraction; MYS‐MMIP, mesoporous yolk‐shell structure magnetic molecularly imprinted polymer; PT‐SPE, pipette tip solid‐phase extraction; SPMIP, magnetic‐surfaced pseudo molecularly imprinted polymer; SS‐MSPME, syringe‐to‐syringe magnetic solid‐phase microextraction.

For the metrics of studies applied to food samples, the values range from 0.27 to 0.64, demonstrating a wide variation in the sustainability of the methods. Most sample preparation methods present moderate environmental efficiency, between 0.3 and 0.4, reflecting common limitations in the procedures applied to food analysis. Only three studies obtained a value greater than 0.5, showing that few methods have characteristics certified with the principles of green chemistry. For the studies that obtained lower metrics, the amount of sample used, the generation of waste, the use of hazardous materials, and the low automation of the methods are noteworthy. Evaluating these values, it is observed that there is still significant room for improving the analytical methods applied to food, adopting strategies that reduce the environmental impact.

##### 3.1.2.3 MIPs in Biological Matrices

The use of MIPs for biological samples with chromatographic detection is more restricted, and only two articles were selected. Below, there are more details on these articles.

Mirzapour and Sadeghi [71] described the synthesis of magnetic MIPs (MMIPs) for SPE and in the controlled release of dextromethorphan at different pH values of simulated biological fluids (7.4 for simulated biological fluid, 1.0 for simulated gastric acid, and 6.8 for simulated intestinal fluid) because of the durability of MIPs under hard conditions, such as in acidic gastrointestinal transit, and targeted and controlled release of the target therapeutic molecule, engineering the architecture by tailoring the degree of cross‐linking. The authors showed that the MMIPs in PBS (phosphate buffered saline, pH 7.4) at 37°C prepared using EGDMA (ethyleneglycol dimethacrylate) as a cross‐linking agent were degraded in 8 days. The relative recoveries of dextromethorphan in spiked serum samples and pharmaceutical products were in the range of 92%–97%. For this study, the score by AGREEprep was 0.31 due to a sample preparation placement ex situ, low sustainability, renewability, and reusability of materials, less than one sample per hour, and six steps or more without automation of the system.

de Oliveira et al. [43] presented a new restricted‐access material combined with an MIP for selective MSPE of estrogens (ethinylestradiol and estradiol) in urine samples from volunteers on hormonal contraceptive use. The protein exclusion test allows us to evaluate whether the material was properly coated with bovine serum albumin (BSA) and can exclude macromolecules in a range from 84.88% to 99.76%. These values indicate that the coated materials exclude the BSA almost completely from the solution (or biological fluids), whereas the uncoated material adsorbs some BSA (protein). The samples were subjected to MSPE, and the results showed that ethinylestradiol was not detected and estradiol was detected at 449.36 ng mL^−1^ in urine samples. The recoveries were assessed by analyzing the spiked human urine samples, ranging from 92.03% to 104.60%, indicating that the developed method was efficiently applied for the selective determination of trace estrogens in complex biological samples. The score obtained by AGREEprep was 0.5, showing an equilibrium between negative and positive aspects. The negative impact comes from Criterion 1, sample preparation placement ex situ, and Criterion 7, four steps of sample preparation without automation. The positive impact came from Criteria 5, 4, 6, and 8, with little waste, size economy of the sample, more than one sample per hour, and low energy consumption per analysis, respectively.

Table 4 shows the characteristics of the above‐mentioned articles [43, 71].

**TABLE 4:** Recent applications of molecularly imprinted polymers in sample preparation methods applying chromatographic separation in biological matrices.

Material	Sample preparation technique	Analytes	Sample	Instrument	Linear range	LOQ	Recovery (%)	AGREEprep	Reference
RA‐MMMIP‐HM‐BSA	MSPE	Estradiol and ethinylestradiol	Urine	HPLC	80–1100 ng mL^−1^	80 ng mL^−1^	92.03–104.6	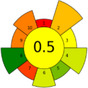	[43]
MMIPs	SPE	Dextromethorphan	Plasma	HPLC‐UV	0.4–200 µg L^−1^	0.4 µg L^−1^	92–97	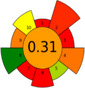	[71]

Abbreviations: AGREEprep, Analytical Greenness Metric for Sample Preparation; HPLC, high‐performance liquid chromatography; RA‐MMMIP‐HM‐BSA, magnetic mesoporous molecularly imprinted polymer double coated with hydrophilic monomer and bovine serum albumin.

The AGREEprep analysis shows that the methods have made significant advances, but there is significant room for improvement in the automation and integration of the use of MIPs in the preparation of biological samples.

### 3.2 MOFs

#### 3.2.1 Fundamentals

Among the materials that have been developed and used in sample preparation techniques, MOFs stand out. The concept of MOFs was presented in the late 1990s by Yaghi, Li, and Li, with the development of a network using cobalt (Co) as the central metal and 1,3,5‐benzenetricarboxylate as the coordinating agent. With the reagents and reactions involved for production, the MOF CoC_6_H_3_(COOH_1/3_)_3_(NC_5_H_5_)_2_…2/3NC_5_H_5_ was successfully obtained by the researchers [72]. From this moment on, new works have emerged and expanded, not only in applications around sample preparation but also in several others.

MOFs are organized structures formed by metal ions or clusters as coordination centers, which are coordinately linked to organic compounds (monodentate, bidentate, or polydentate), generating a continuous and ordered network [73, 74]. Due to this broad concept, the versatility of production of different MOFs allows them to have different physical–chemical characteristics, among them voluminous surface areas, adjustable and generally high porosities, and structures that can be flexible to rigid, creating crystalline networks, in addition to allowing different modifications and reactions to be performed on their surfaces, leading to different applications and interactions with various compounds (inorganic/organic) [75, 76, 77].

The study of MOFs has its roots in inorganic chemistry but has developed into a new field. Furthermore, MOFs are constructed from organic bridging ligands that remain intact throughout the synthesis. The synthesis of MOFs generally involves the combination of metal salts with organic ligands under specific conditions that favor the formation of the desired structure, each of which directly influences the final properties of the material. There are different synthetic routes to produce MOFs, among which the ionothermal method, which uses ILs as solvents in the process and structuring agents, stands out. Of interest too are hydrothermal or solvothermal methods that consist of the reaction of metal precursors with organic ligands in aqueous or organic solvents under high temperatures and pressures in sealed autoclaves, which allows the formation of high‐quality crystals and is widely used in the synthesis of MOFs. Another method is using DESs as alternatives to the usual solvents for the production of MOFs as more environmentally friendly proposals. Surfactant‐thermal processes are used to control the size and morphology of MOF crystals during thermal synthesis and mechanosynthesis or mechanochemical synthesis. These involve grinding the metal precursors and ligands in a ball mill, promoting the reaction in the solid state without the use of solvents [78, 79, 80, 81].

Among the different fields of applications, there are catalysis [82, 83], biomedicine [84, 85, 86], gas storage and separation [87, 88], energy conversion [89, 90], conductivity [91, 92], sensing in liquid or solid media [93, 94, 95], water treatments [96, 97, 98], capture and adsorption of compounds [99, 100], and other areas. In the area of sample preparation, there is a growing interest in the development and applications of different techniques due to their unique physical–chemical properties and versatility in different compositions, which can be selective and specific materials for different purposes, with a gradual increase in the extraction of compounds in environmental and food matrices and in a few applications in the biological area in recent years [101, 102, 103, 104, 105, 106, 107].

MOFs are promising materials in analytical chemistry due to their unique properties. Their use meets the principles of GAC, being effective in extraction/microextraction, sensors, and chromatography. However, their sustainability depends on design, synthesis, and toxicity assessment. There is potential for several applications, but research is needed on toxicity and low‐cost alternatives, encouraging collaboration between materials science and analytical chemistry [16]. In the following section, some notable applications of MOFs used in sample preparation techniques and articles published in the literature are presented, with critical reviews in green metrics and methodology development for different samples.

#### 3.2.2 Recent Applications of MOFs in Sample Preparation

##### 3.2.2.1 MOFs in Environmental Samples

MOFs stand out in applications for monitoring and adsorption of pollutant and toxic compounds in environmental matrices. Among their physicochemical properties, MOFs are a promising tool for applications such as water treatment, emission control, and environmental decontamination.

Guerra‐Martín et al. proposed a material based on MOF, using iron (Fe) as the central metal [108]. One of the most notable aspects of this approach is the innovative synthetic route employed. Instead of conventional methods that require high temperatures or pressures, the authors used a precipitation method under optimized conditions at room temperature to synthesize the MOF. Furthermore, they minimized the use of organic solvents, favoring water as the primary solvent and a basic sodium hydroxide solution. Another intriguing aspect of their method was the sustainable approach to sample preparation. Recycled yogurt containers, thoroughly cleaned, were repurposed to create a dispersion of the MOF. In addition, cellulose supports were cut and prepared for the material to be applied using the TF‐SPME technique. This system using MIL‐100(Fe) material was then used to extract seven compounds commonly found in personal care products. Through statistical analysis, the authors optimized, validated, and successfully applied the developed methodology to environmental samples (swimming pool water) and cosmetics (micellar water), with LODs ranging from 2.5 to 7.5 µg L^−1^ and precision lower than 11%. The sustainability of the method was highlighted by using specific tools and comparing it to a traditional method based on SPE. The comparison showed improvements, with higher scores and more regions marked by green indicators, reflecting the greener and more environmentally friendly nature of the new method. Considering the previous inference, the AGREEprep gave a score of 0.40 for this methodology. Despite this below‐average score, it should be considered that in the criteria of material reuse (Criterion 3), waste (Criterion 4), quantity of samples per hour (Criterion 6), and use of manual systems for preparation (Criterion 7), the scores are low, which affects the final score.

In a recent collaborative study between Iran and Turkey, Pezhhanfar et al. pioneered the use of a heterometallic MOF, incorporating two metal centers: sodium (Na) and bismuth (Bi) [109]. This material was applied to the extraction of phthalate esters and adipates from various water sources, including tap, well, river, and surface water. Notably, the authors employed 2,2‐dimethoxypropane as a ternary solvent for the in situ desorption process. One significant aspect of using this solvent is that a small portion remained in the MOF particles after the dispersive micro‐solid‐phase extraction (DµSPE), whereas another portion was converted into smaller structured solvents, such as acetone and methanol. Importantly, only a minimal volume of the solvent (500 µL) was used in the process. The developed methodology demonstrated excellent performance for the target analytes, with LODs and LOQs being 0.66–1.20 and 2.18–3.97 µg L^−1^, respectively. From a sustainability perspective, the method is advantageous due to its low consumption of solvents and materials, its versatility in application, and the reduced preparation and chromatographic analysis times required. Despite these positive points, when evaluated using the AGREEprep, a score of 0.38 was obtained, which was mainly caused by the process being completely manual, from preparation to the moment of injection into the chromatographic equipment. The materials, despite being in small quantities, are not reusable or renewable (Criterion 3), and it is an ex situ methodology (Criterion 1); therefore, this affects the green aspect of the method developed.

Another noteworthy application in the environmental field involved the development and use of MOF composed of a spherical assembly of carbon nanorods with hierarchical porosity. Tao and his research group designed this material as an alternative coating for SPME [110]. To demonstrate the effectiveness of these new carbon‐based fibers, they applied the technique to the detection of nitrated polycyclic aromatic hydrocarbons (NPAHs), which are hazardous environmental pollutants with known adverse effects on both ecosystems and human health. This new MOF offered several advantages, including a high surface area and a pore structure ranging from micro to macro sizes, which facilitated efficient mass transfer of analytes from the sample matrix to the extraction phase. Additionally, the MOF's hydrophobic groups enhanced its interaction with the analytes. The SPME fiber, integrated into a GC‐electron capture detector (ECD) system, exhibited excellent physical, chemical, and thermal stability, allowing it to be reused across multiple experimental cycles. The method was validated with strong performance results (LODs ranging from 0.4 to 0.5 ng L^−1^) and successfully applied to three different water samples (tap, surface, and groundwater). The sustainability of the methodology was assessed using AGREEprep software, and a score of 0.57 was obtained, which confirmed that the method aligned with several principles of GAC, highlighting its environmental benefits, including low consumption of energy (Criterion 8), no toxic materials during preparation (Criterion 2), ensuring the health of the analyst (Criterion 10), and the materials can be reused several times (Criterion 3), ensuring less waste generation (Criterion 4). Other applications of MOFs in sample preparation for environmental matrices are presented in Table 5 [105, 108–129].

**TABLE 5:** Recent applications of metal–organic frameworks (MOFs) in sample preparation methods applying chromatographic separation in environmental matrices.

Material	Sample preparation technique	Analytes	Sample	Instrument	Linear range	LOQ	Recovery (%)	AGREEprep	Reference
Cu–MOF/COF	SPME	6 PAHs	Soil	GC‐FID	0.5–1000 ng L^−1^	0.5–1.0 ng L^−1^	88.71–104.08	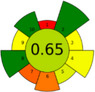	[105]
MIL‐100(Fe)/PS	TF‐SPME	Triclosan, benzophenone, enzacamene, ethylparaben, propylparaben, and butylparaben	Swimming pool water	HPLC–DAD	8–1000 µg L^−1^	—	—	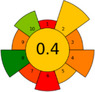	[108]
Na‐Bi‐MOF	DµSPE	Di‐2‐ethylhexyl adipate, 2‐ethylhexyl phthalate, di‐iso‐butyl phthalate, and di‐*n*‐butyl phatalate	Tap, well, surface, and river waters	GC‐FID	2.18–500 µg L^−1^	2.18–3.97 µg L^−1^	86–117	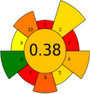	[109]
HP‐MOF‐C	SPME	8 NPAHs	Tap, surface, and groundwater	GC‐ECD	5.0–10 000 ng L^−1^	1.3–16.7 ng L^−1^	83.7–119.4	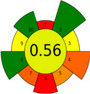	[110]
MIL‐100(Fe)@MIL‐53(Fe)	DµSPE	Clobazam, clonazepam, and oxcarbazepine	Well, tap, and river water	GC–MS	0.1–368 µg L^−1^	0.1–0.2 µg L^−1^	92.5–97.6	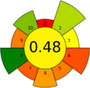	[111]
Fe_3_O_4_NPs@SiO_2_@Bimetallic Ni‐Zn‐MOF	DµSPE	Fenitrothion, chlorpyrifos, and diazinon	Tap, well, dam, and river waters	GC‐FID	0.17–259 µg L^−1^	0.17–0.25 µg L^−1^	91.2–97.7	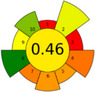	[112]
ZIF‐67@NiCo‐LDHs	SPME	9 PAHs	River water	GC–MS	0.0001–100 µg L^−1^	0.08–2.7 ng L^−1^	—	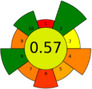	[113]
PAN/MIL‐53(Al)@SBA‐15/BiPy^2+^2Cl^−^	SPME	Benzene, toluene, ethylbenzene, trimethylbenzene, styrene, and *p*‐xylene	River and wastewater	GC‐FID	0.01–250 µg L^−1^	0.003–0.0036 µg L^−1^	92–101	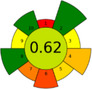	[114]
CIM‐80(Al)	SPME	14 Phthalates	Bottled water	UHPLC–MS/MS	0.04–1000 µg L^−1^	0.04–1.50 µg L^−1^	70–107	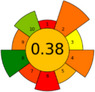	[115]
ZIF‐8@monolith	SPME	Norfloxacin, fleroxacin, danofloxacin, enrofloxacin, and sarafloxacin	Tap, river, and wastewater	HPLC‐FLD	0.001–5.0 µg L^−1^	0.00048–0.0018 µg L^−1^	80.1–120	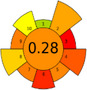	[116]
Zr/N‐OMC	SPME	2‐Clorophenol, 4‐methylphenol, 2,6‐dimethylphenol, *o*‐nitrophenol, 2,4‐dichlorophenol, and 2,4,6‐trichlorophenol	Pearl river and pond waters	GC–MS	10–20 000 ng L^−1^	—	84.5–108	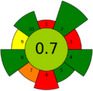	[117]
ZIF‐8@ZIF‐67	SPME	Dimethyl, diethyl, di‐*n*‐butyl, benzyl butyl, and di isobutyl phthalates	Drinking and mineral waters	GC–MS	0.03–30 ng mL^−1^	0.03–0.07 ng mL^−1^	84–106	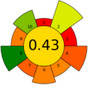	[118]
MOF‐74‐C	SPME	2‐Chlorophenol, 2,4,6‐trichloroanisole, 2‐isobutyl‐3‐methoxypyrazine, thiophenol, and 4‐methylthiophenol	Freshwater, tap, and wastewater	GC–MS	0.005–100 µg L^−1^	0.00003–0.3 µg L^−1^	90.1–107.3	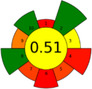	[119]
MOF‐199	SPME	Benzene, toluene, ethylbenzene, *m*‐xylene, and *p*‐xylene	Air	GC–MS	5–100 µg m^−3^	0.09–0.31 µg m^−3^	73–108	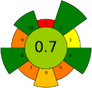	[120]
2DTP/MIL‐101‐Cr	SPME	6 BTEX and 6 PAHs	Soil	GC‐FID	0.23–9000 ng g^−1^	0.23–16.9 ng g^−1^	80.4–108	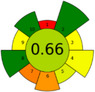	[121]
N‐CNTC	SPME	7 Polychlorinated biphenyls	River water	GC–MS	0.3–1000 ng L^−1^	0.33–0.72 ng L^−1^	—	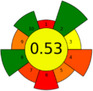	[122]
ZIF‐8‐like microporous shell	SPME	5 Polychlorinated biphenyls	Rainwater, pond, and river waters	GC–MS	0.05–1000 ng L^−1^	0.0057–0.11 ng L^−1^	84.5–117.1	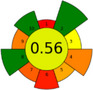	[123]
YS‐NH_2_‐UiO‐66@CoZn‐ZIF	SPME	6 Pesticides	River water	HPLC‐UV	0.241–500 µg L^−1^	0.241–1.891 µg L^−1^	85.1–103.8	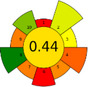	[124]
Zn‐MOF‐NH_2_/COF	SPME	6 PAHs	Soil	GC‐FID	1–20000 ng g^−1^	—	91.1–110.2	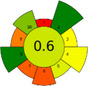	[125]
MOF‐199@MON	SPME	BTEX	Tap and lake water	GC–MS	0.5–500 µg L^−1^	0.04–0.12 µg L^−1^	71.0–113	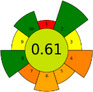	[126]
H‐ZIF‐8@Zn‐MOF‐74	SPME	6 Organic nitrogen pesticides	Waste water	HPLC‐FLD	0.072–1000 µg L^−1^	0.072–1.406 µg L^−1^	83.86–111.8	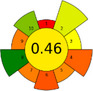	[127]
PVA@UiO‐66	SPE	Diethyl phthalate, dimethyl phthalate, and benzyl butyl phthalate	Tap, river, well, and wastewater	GC‐FID	0.05–100 µg L^−1^	0.05–0.2 µg L^−1^	91.2–99.2	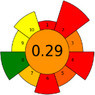	[128]
Cu–MOF/GO	SPE	4 PAHs	Tap, river, well, and wastewater	GC‐FID	0.001–30 µg L^−1^	0.001–0.006 µg L^−1^	95.1–99.5	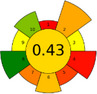	[129]

Abbreviations: 2DTP/MIL‐101‐Cr, MOF hybrid with COF; Cu–MOF/COF, MOF combined with COF; Cu–MOF/GO, Cu‐based metal–organic framework/graphene oxide; Fe_3_O_4_NPs@SiO_2_@bimetallic Ni‐Zn‐MOF, bimetallic MOF with magnetic nanoparticles; HP‐MOF‐C, MOF‐derived hierarchical carbon; H‐ZIF‐8@Zn‐MOF‐74, a urchin‐shaped hollow MOF; MIL‐100(Fe)/PS, cellulose supported MOF/recycled polystyrene; MIL‐100(Fe)@MIL‐53(Fe), hybrid core–shell MOF; MOF‐199@MON, MOF‐199 in microporous organic networks; MOF‐74‐C, MOF carbonized; Na‐Bi‐MOF, bimetallic MOF; N‐CNTC, MOF with nitrogen‐doped carbon nanotubes cages; PAN/MIL‐53(Al)@SBA‐15/BiPy^2^+2Cl^−^, polyacrylonitrile‐MOF modified with 4,4′‐bipyridine silica nanofibers; PVA@UiO‐66, electrospun composite polyvinyl alcohol/zirconium‐based MOF nanofiber coating; YS‐NH_2_‐UiO‐66@CoZn‐ZIF, yolk‐shell structure; ZIF‐67@NiCo‐LDHs, bimetallic MOF protected by layered double hydroxides nanotubes; ZIF‐8@monolith, MOF with monolith composite; ZIF‐8@ZIF‐67, MOF on MOF composite; ZIF‐8‐like microporous shell, MOF with hollow carbon nanobubbles (HCNBs) with ultrathin micropores shell; Zn‐MOF‐NH_2_/COF, an MOF and COF combinate hybrid material; Zr/N‐OMC, MOF with ordered mesoporous carbons.

Considering the methodologies presented in Table 5 involving the application of MOFs in environmental samples, there is a wide range of values obtained by the AGREEprep, ranging from 0.28 to 0.70, with an overall average of 0.52. This shows that the methods have been developed with green chemistry principles, mainly in the reduction of waste generation, reuse of MOFs in different sample preparation techniques, and increase in the analytical frequency of samples per hour.

##### 3.2.2.2 MOFs in Food Samples

In the context of food samples, MOFs have been widely used to detect possible compounds that may migrate from packaging to food or are used in crops for conservation and storage and that may become contaminants. Thus, their use has been interesting for food safety and quality control.

**FIGURE 2:**
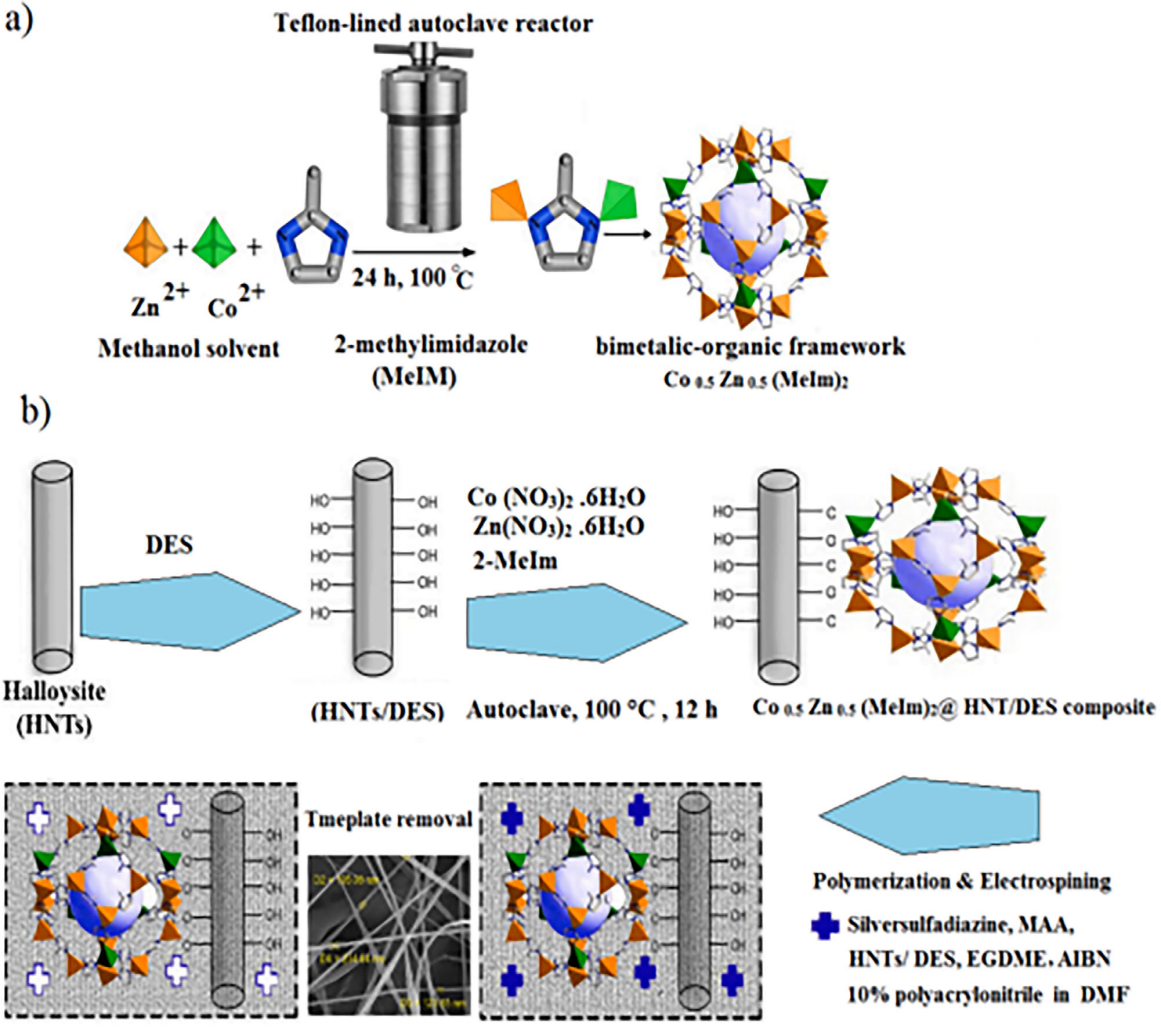
Illustrative scheme of the steps involved in the synthesis of the complete material, comprising (A) Bimetallic MOF structure and (B) electrospun thin film nanofibers based on bimetallic MOF structure composite (Co_0.5_Zn_0.5_(MeIm)_2_@HNT/DES/MIPs). DES, deep eutectic solvent; EGDMA, ethyleneglycol dimethacrylate. *Source*: Reproduction authorized according to Mirzajani and Kha [130].

In 2024, Mirzajani and Kha developed a composite material, where the bimetallic MOF of cobalt (Co) and zinc (Zn) was produced and used to synthesize a new extraction phase for the TF‐SPME technique, using in its composition DES, functionalized halloysite nanotubes (HNT), and MIPs [130]. This new material was created in individual steps, but the final production used the electrospun nanofiber technique. Figure 2 shows an illustration made by the authors to demonstrate the synthetic route in a complete manner, from the production of the bimetallic structure of the MOF used to its modification to obtain the final material used in sample preparation. In this work, the MOF enters as a support component with high surface area particles, and the addition of HNT and DES allows the presence of larger adsorption sites and functional groups for interaction. The extraction phase used in the TF‐SPME technique was applied for the extraction of sulfonamides, using chemometric metrics for optimization and development of the methodology, and to determine these analytes in different food matrices, including animal milk, egg, and chicken meat. For validation, good analytical performance was obtained, with an LOD of 0.003 µg L^−1^ and LOQ of 0.01 µg L^−1^. The authors obtained satisfactory results in both matrices applied, as well as demonstrating that the developed method can be an important tool in the monitoring of these sulfonamides, because they are used as antibiotics in the treatment of animals and can migrate to foods. However, when the method's green impacts were assessed, according to AGREEprep, a score of 0.37 was obtained. This score is lower than an environmentally friendly method, because some toxic materials are employed for production and use in sample preparation (Criterion 2), a long preparation time, considering an average of 1.5 samples per hour (Criterion 6), and the process is carried out in different, distinct stages manually, which makes the method time‐consuming (Criterion 7). The positive points are the small number of samples (Criterion 5) and the reuse of materials, such as MOFs, several times (Criterion 3).

In another recent collaborative project between Iran and Canada, Javanmardi et al. synthesized three different MOFs to be used as the extraction phase for an SPME, created from the sol–gel technique and 3‐aminopropyltriethoxysilane as a ligand, to perform the determination of organophosphate pesticides in tomato samples [131]. Amino‐based MOFs (MIL‐101‐NH_2_) with a chromium (Cr) as metal center were modified with acyl and ethyl groups. Studies have shown that MIL‐101‐NH_2_‐COCH_3_ had the best extractions of pesticides, because they have many polar groups and had better interactions with the acyl group added to the MOF. The co‐workers obtained good analytical responses and validation parameters for the four established analytes, with LODs in the range of 0.2–1.0 ng kg^−1^ and precisions below 13% and 15% for intraday and interday, respectively. In addition, in the sample preparation, they did not use solvents to perform the extraction/desorption procedure, with little waste generation. By applying the method to three different tomato samples, the authors detected the presence of three of the four analytes, demonstrating the importance of monitoring such compounds, because the health problems they cause in living beings are already established. This method presented a value of 0.62 in the AGREEprep tool, considered a score that indicates a method with green and environmentally friendly principles, mainly with regard to the non‐use of toxic materials (Criterion 2), small quantities of samples used (Criterion 5), and that guarantees the operation of the method in a safe manner for the operator (Criterion 10).

Another use of the SPME technique was carried out by Hasani et al. in 2023, where the authors used a copper (Cu) MOF as a new in situ fabricated Cu@porous carbon fiber derived from the copper benzene‐1,4‐dicarboxylate@pencil graphite (Cu‐BDC MOF@PG) structure [132]. In this study, the nanoporous structure material was electrodeposited directly on the surface with the graphitized region of a pencil as a support, and, after this process, fibers were produced using this system. The SPME technique, used in direct immersion mode, was applied to determine imidacloprid and acetamiprid, two potential pesticides of the neonicotinoid class, which are regulated in several countries. Their residues can be found in food from agriculture, and they need to be monitored, because they potentially have several harmful effects if in high concentrations. Good validation results were obtained by the researchers, in addition to demonstrating that the MOF‐SPME‐HPLC‐UV method developed is simple to use, cost‐effective, and presents good recovery (87%–109%) and repeatability (≤3.70%), with applications in different food matrices, with juices being produced from melon, cucumber, tomato, and pear, obtaining satisfactory recovery and precision results. Evaluating the sustainable aspects using the AGREEprep tool, a score of 0.45 was defined, showing a tendency of the method, in some points, to be chemically green, with the strong points being the low consumption of materials (Criterion 5), low generation of waste (Criterion 4), and energy consumption (Criterion 8) during the process, and few materials used are toxic to the operator (Criterion 10). Other applications of MOFs in sample preparation for food matrices are presented in Table 6 [116, 118, 128, 130–142].

**TABLE 6:** Recent applications of metal–organic frameworks (MOFs) in sample preparation methods applying chromatographic separation in food matrices.

Material	Sample preparation technique	Analytes	Sample	Instrument	Linear range	LOQ	Recovery (%)	AGREEprep	Reference
ZIF‐8	SPME	Norfloxacin, fleroxacin, danofloxacin, enrofloxacin, and sarafloxacin	Honey	HPLC‐FLD	0.001–10 µg L^−1^	0.00014–0.0011 µg L^−1^	80.1–117	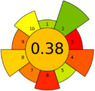	[116]
Fe_3_O_4_@ZIF‐8@ZIF‐67	SPME	Dimethyl phthalate, diethyl phthalate, di‐*n*‐butyl phthalate, benzyl butyl phthalate, and di‐isobutyl phthalate	Orange and grape	GC–MS	0.03–30 ng mL^−1^	0.03–0.07 µg L^−1^	84.0–106.0	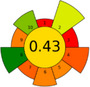	[118]
PVA@UiO‐66	SPE	Diethyl phthalate, dimethyl phthalate, and benzyl butyl phthalate	Milk	GC‐FID	0.05–100 µg L^−1^	0.05–0.2 µg L^−1^	89.5–96.9	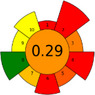	[128]
Co_0.5_Zn_0.5_(MeIm)_2_@HNT/DES/MIPs	TF‐SPME	Sulfacetamide, sulfamethoxazine, sulfamethoxazole, and silver sulfadiazine	Milk, egg, and chicken meat	HPLC‐UV	0.01–50 µg L^−1^	0.011–0.013 µg L^−1^	95.9–101	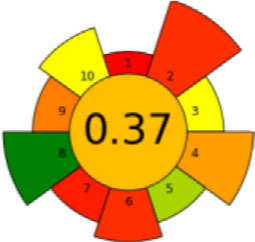	[130]
MIL‐101‐NH_2_	SPME	Fenthion, profenofos, ethion, and phosalone	Tomato	GC–MS	1–1000 ng kg^−1^	1–5 ng kg^−1^	80–91	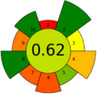	[131]
Cu‐BDC MOF@PG	SPME	Acetamiprid and imidacloprid	Melon, cucumber, tomato, and pear	HPLC‐UV	0.5–200 µg L^−1^	0.5–1.0 µg L^−1^	87–109	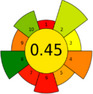	[132]
NH_2_‐MIL‐53(Al) incorporated poly(AAPBA/MAA‐co‐EGDMA)	SPME	Sulfadiazine, sulfathiazole, sulfamerazine, sulfamethazine, sulfamonomethoxine, sulfamethoxazole, sulfisoxazole, and sulfadimethoxine	Chicken and fish	UHPLC–MS/MS	0.015–25.0 µg L^−1^	4.15–14.2 ng L^−1^	85.7–113	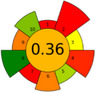	[133]
C‐(C_3_N_4_@MOF)	SPME	Phorate, dimethoate, diazinon, disulfoton, iprobenfos, parathion methyl, malathion, chlorpyrifos, parathion, isocarbophos, phenthoate, profenofos, ethion, and triazophos	Apple, peach, pear, nectarine, plum, cabbage, lettuce, pakchoi, and oilseed rape	GC–MS	0.69–3000 ng g^−1^	0.69–22.5 ng g^−1^	82.6–118	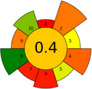	[134]
UiO‐66‐NH_2_	SPME	5 PAHs	Milk	GC‐FID	0.2–50 ng mL^−1^	0.07–0.24 ng mL^−1^	85.43–115.8	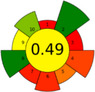	[135]
MIL‐101(Cr)NH_2_‐polyacrylonitrile	SPME	Flumequine, nalidixic acid, sulfadimethoxine, sulfaphenazole, tilmicosin, and trimethoprim	Fish muscle	LC–MS/MS	10–50000 ng L^−1^	0.6–15.5 ng L^−1^	—	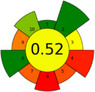	[136]
MIL‐101(Cr)	TF‐SPME	Sulfadiazine, sulfathiazole, sulfamerazine, sulfadimidine, sulfamethoxazole, and sulfisoxazole	Honey, pork, chicken, and milk	HPLC–DAD	10.0–200.0 ng mL^−1^	8.0–14.5 ng mL^−1^	91.4–107.9	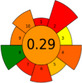	[137]
ZIF‐8@GO	TF‐SPME	Spiramycin, tilmicosin, oleandomycin, tylosin, kitasamycin, erythromycin, josamycin, roxithromycin, lincomycin, and clindamycin	Honey	UHPLC–MS/MS	0.2–200 µg L^−1^	0.4–1.0 µg L^−1^	68–107	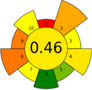	[138]
PAN/ϒ‐CD‐MOF	TF‐SPME	Clodinafop‐propargyl, fenoxaprop‐*p*‐ethyl, oxyfluorfen, and pendimethalin	Wheat, rice, and barley cereals	HPLC‐UV	2.5–1250.0 ng mL^−1^	2.5–7.5 ng mL^−1^	92.9–106.1	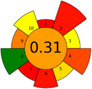	[139]
3D HZIF‐67@Co‐Al LDH	TF‐SPME	Caffeine	Coffee and beverage	HPLC‐UV	1–200 µg L^−1^	1.1 µg L^−1^	96–98	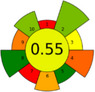	[140]
NH_2_‐MIL‐88@PCN‐224	SPME	5 PAHs	Milk	GC‐FID	1–200 µg L^−1^	0.010–0.067 µg L^−1^	92.15–104.64	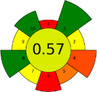	[141]
Ni‐Co MOF/Zn‐NTA	SPME	Difenoconazole, hexaconazole, and triticonazole	Orange, mandarin, lemon, apricot, cherry juices, and lemon	HPLC–DAD	—	5.3–8.0 µg L^−1^	87–92	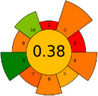	[142]

Abbreviations: 3D HZIF‐67@Co‐Al LDH, bimetallic MOF with double hydroxide nanosheets to three‐dimensional hierarchical ZIF; C‐(C_3_N_4_@MOF), MOF with nitrogen‐doped porous carbon; Co_0.5_Zn_0.5_(MeIm)_2_@HNT/DES composite/MIPs, combination with MOF, halloysite nanotubes, DES, and MIP; Fe_3_O_4_@ZIF‐8@ZIF‐67, MOF on MOF based with nanoparticles; MIL‐101(Cr)NH_2_‐polyacrylonitrile, MOF modified with amino groups and polymer based; NH_2_‐MIL‐53(Al) incorporated poly(AAPBA/MAA‐co‐EGDMA), MOF incorporated in poly(3‐acrylamidophenylboronic acid/methacrylic acid‐co‐ethylene glycol dimethacrylate); NH_2_‐MIL‐88@PCN‐224, amino composite MOF with porous coordination network formed by Zr (IV) and tetrakis (4‐carboxyphenyl)porphyrin; Ni‐Co MOF/Zn‐NTA, bimetallic MOF with nitrilotriacetic acid forming nanoflowers; PAN/ϒ‐CD‐MOF, nanofibers of crosslinked polyacrylonitrile/ϒ‐cyclodextrin‐MOF; UiO‐66‐NH2, MOF/COF composite; ZIF‐8@GO, MOF with graphene oxide.

For applications in food samples, the methods with MOFs presented in Table 6 show AGREEprep values from 0.29 to 0.62, with an overall average of 0.43. In these applications presented, an overall average of the works indicates that the tendency to apply green principles has been growing, considering that few works present values well above this average for this type of application.

##### 3.2.2.3 MOFs in Biological Samples

MOFs have a variety of emerging applications, and, depending on the biological area, they have been extensively used in the monitoring of drugs, metabolites, and other compounds that are related to poisoning, diseases, and other adversities.

In a recently published article, Kharazmi et al. synthesized and characterized a trivalent MOF using nickel (Ni), cobalt (Co), and zinc (Zn), which was used in the manufacture of nanofibers using the electrospinning method and was modified with polyvinyl alcohol (PVA), polyacrylic acid (PAA), and GO support, forming the final nanocomposite named PVA/PAA/MOF NiCoZn‐layered double hydroxide (LDH)@GO [143]. The combination of these components allowed the generation of a porous material with several functional groups for the extraction of opioid drugs and analgesics, used in the TF‐SPME technique, where the produced fibers generate a network of electrospun nanofibers compacted into a film, which were used to extract these target compounds from biological matrices of human plasma and urine. The authors performed the appropriate characterizations of the material and optimizations of the method, obtaining desirable parameters for the extraction of these compounds. Good merit parameters were obtained, as well as high enrichment factors. The production of nanofibers is done by a low‐cost method, with little waste generation, as well as the use of small amounts of sample and toxic organic solvents. Therefore, the methodology presented in this article was effective for the extraction and determination of seven drugs in studied samples, with satisfactory analytical parameters of merit considering LODs of 0.10–0.15 µg L^−1^ and LOQs from 0.3 to 0.5 µg L^−1^. Using AGREEprep, a score of 0.25 is obtained, which is considered a low value for an environmentally friendly method. Because the methodology uses relatively high amounts of toxic materials (Criterion 2), low reuse of materials (Criterion 3), large amounts of samples and solvents (Criterion 4), and no automation of the preparation steps involved (Criterion 7), it presents this relatively low score despite its great advantage for the desired application.

In an unprecedented proposal, Shokrollahi et al. developed a methodology using the SPME technique where the coating was produced using the in situ electrosynthesis technique to form a structure of the copper (Cu)‐based MOF material. In this study, the produced fibers were treated with different compounds, the main one being the ligand 1,2,4,5‐benzenetetracarboxylate acid [144]. The methodology was developed for the extraction of free methamphetamine in urine samples, and with the treatments performed on the material by the authors, it allowed different interactions to be possible such as π–π stacking, H‐bonding, and hydrophobic. Thus, with the optimizations properly proposed and performed, good validation parameters were possible (LOD of 0.1 ng mL^−1^), and they were then applied in two samples, with good recovery results in both (85.0%–102.5%). This showed the stability of application in different matrices, because in the case of biological samples, it varies from individual to individual and from day to day. The method proved to be promising, as the authors showed that the fibers produced have good reproducibility, and, as it is a method with the SPME technique, it eliminates the use of organic solvents in sample preparation, generating little waste, and presenting green aspects. In this method, the AGREEprep tool was applied, obtaining a score of 0.62. This indicates a good correction with green principles for sample preparation applied in the methodology, because the authors did not use toxic materials in the process (Criterion 2), used low amounts of samples and solvents (Criterion 5), and ensured safe operation for the analyst who is applying the method developed with the MOF.

In order to apply a developed extraction method for ibuprofen, simvastatin, and ranitidine from rat plasma, Liu et al. performed a combination of two sample preparation techniques, liquid‐phase microextraction (LPME) and SPME, forming a new configuration, liquid–liquid–solid membrane microextraction (LLSMME) [145]. In this case study, the authors synthesized the MOF and produced a homogeneous zeolitic imidazolate framework‐8 mixed matrix membrane (ZIF‐8‐MMM), preparing it in situ on the inner surface of the hollow fiber membrane. This combination of technique and extractant material allowed the effective determination of these three drugs when they were extracted from laboratory rats via blood plasma, with good validation results, namely, an LOD of 2–3 ng mL^−1^, reproducibility ranging from 97.42% to 103.8%, and high enrichment factors in the values of 87.3%–112.6%. One of the advantages was the useful life of this system, making it possible to recycle the material up to 30 times in experimental cycles and maintaining its significantly high and invariable efficiency. The quantity of solvents used was microliters, as well as the amount of sample required to perform the procedure, generating little waste. Thus, the developed method presented promising results as a green alternative to other bioanalysis methods, with effective extraction, determination, and identification of target compounds and meeting the precepts of GAC. In this last MOF methodology described, the score obtained by AGREEprep was 0.48. This is an average score that indicates some principles that are in‐line with green chemistry. The main points of this method are the low use of toxic materials (Criterion 2), low volume of samples/solvents used (Criterion 5), and the multiple reuses of some materials, including the MOF used (Criterion 3). Other applications of MOFs in sample preparation for biological matrices are presented in Table 7 [140, 143–151].

**TABLE 7:** Recent applications of metal–organic frameworks (MOFs) in sample preparation methods applying chromatographic separation in biological matrices.

Material	Sample preparation technique	Analytes	Sample	Instrument	Linear range	LOQ	Recovery (%)	AGREEprep	Reference
3D HZIF‐67	TF‐SPME	Caffeine	Urine	HPLC‐UV	1–200 µg L^−1^	1.2 µg L^−1^	88–91	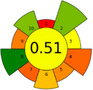	[140]
NiCoZn‐LDH@GO/carbon cloth	TF‐SPME	Caffeine, tramadol, codeine, hydrocodone, naloxone, noscapine, and celecoxib	Urine and plasma	HPLC‐UV	0.3–1000 µg L^−1^	0.1–0.5 µg L^−1^	86–97	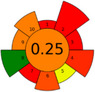	[143]
Cu‐based MOF	SPME	Methamphetamine	Human urine	GC‐FID	0.90–1000 ng mL^−1^	0.9 ng mL^−1^	85.0–102.5	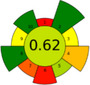	[144]
ZIF‐8‐MMM	LLSMME	Ibuprofen, simvastatin, and ranitidine	Rat plasma	UHPLC–MS/MS	10–200 ng mL^−1^	NI	97.42–103.8	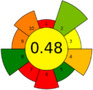	[145]
Magnetized nanokerattin@NH_2_‐ZIF‐7;MKNZ	DµSPE	Celecoxib	Human plasma, breast milk, and urine	HPLC‐UV	0.1–10 ng mL^−1^	0.10 ng mL^−1^	95.25–99.40	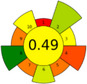	[146]
CoFe_2_O_4_ magnetic nanoparticles	M‐SA‐DSPE	Cortisol and cortisone	Human saliva	LC–MS/MS	0.3–20 ng mL^−1^	0.060–0.097 ng mL^−1^	86–111	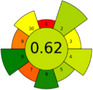	[147]
NH_2_‐MIL‐125@RAMIPs	SPE	Gatifloxacin	Bovine serum	HPLC‐UV	0.01–100 µg mL^−1^	—	97.8–105.6	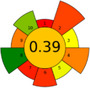	[148]
IRMOF‐3@MLDH	SPE	Vitamin D	Blood samples	LC–MS/MS	5–2000 ng mL^−1^	4.5 ng mL^−1^	95.2–101	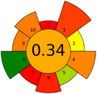	[149]
Polyfam/Co‐MOF‐74	TF‐SPME	Sorafenib, dasatinib, and erlotinib hydrochloride	Urine, plasma, and serum	HPLC‐UV	0.1–1500 µg L^−1^	0.1–0.5 µg L^−1^	86.8–99.3	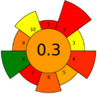	[150]
Mo(PDA)(NO)(µ‐O)MoO_3_]·1.42H_2_O·0.58C_2_H_5_OH}*_n_*	DµSPE	Amitriptyline, nortriptyline, imipramine, and sertraline	Human plasma	HPLC‐UV	0.1–500 ng mL^−1^	—	94.9–102	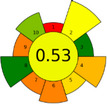	[151]

Abbreviations: 3D HZIF‐67, MOF with hierarchical 3D ZIF; CoFe_2_O_4_ magnetic nanoparticles, MOF with nanoparticles; IRMOF‐3@MLDH, magnetic layered double hydroxide/MOF composite; magnetized nanokerattin@NH_2_‐ZIF‐7;MKNZ, MOF functionalized with amino groups and nanokeratin; Mo(PDA)(NO)(µ‐O)MoO_3_]·1.42H_2_O·0.58C_2_H_5_OH}*_n_*, molybdenum‐based coordination polymer; M‐SA‐DSPE, modified magnetic‐based solvent‐assisted dispersive solid‐phase extraction; NH_2_‐MIL‐125@RAMIPs, MOF amino based with restricted access MIP; NiCoZn‐LDH@GO/carbon cloth, MOF trimetallic with polyvinyl alcohol, polyacrylic acid, and graphene oxide; polyfam/Co‐MOF‐74, MOF with polyfam 620.

In this last section on MOFs, the applications presented in biological matrices of the developed methods presented AGREEprep scores ranging from 0.25 to 0.62, with an average of 0.44. The methods have been presenting improvements with environmentally friendly aspects, and there has been growth in terms of the reuse of MOFs in sample preparation techniques, as alternative materials to commercial ones with high performance.

### 3.3 Conductive Polymers

#### 3.3.1 Fundamentals

Since the discovery by Shirakawa et al. [152] that polyacetylene could be made conductive like a metal, the development of CPs has rapidly accelerated due to their significant scientific and technological importance. CPs represent a unique class of materials that exhibit electrical, optical, and magnetic properties similar to metals and semiconductors, while also providing the benefits of low density and the versatility of polymers [153]. These materials are characterized by a conjugated polymer chain consisting of alternating double and single bonds along the main chain. In their non‐doped state, the electrical conductivity of conjugated polymers is comparable to that of insulating polymers [154]. However, through various doping processes, these conjugated polymers can be transformed into a doped state, resulting in significant alterations to their electrical, optical, and magnetic properties. For example, the electrical conductivity of CPs can increase by several orders of magnitude, shifting from an insulating level (10^−15^ S cm^−1^) to that of a conductive material (10^3^ S cm^−1^) [155].

The highest electrical conductivity value (10^5^ S cm^−1^) has been observed in iodine‐doped polyacetylene [156]. However, the practical applications of polyacetylene are limited due to its high instability in air and inferior mechanical properties [157]. To address these challenges, researchers have explored air‐stable CPs such as polyaniline (PANI), polypyrrole (PPy), poly(thiophene) (PTh), and poly(3,4‐ethylene dioxythiophene) (PEDOT) [156]. These materials offer several advantages, including environmental stability, ease of synthesis, and customizable physicochemical properties, which can be controlled by adjusting the doping levels [158, 159].

There are several methods for producing PANI, PPy, PTh, and PEDOT in their doped states, including electrochemical and chemical oxidative polymerization of their respective monomers [156]. Among these techniques, chemical oxidative polymerization is preferred for its simplicity and scalability. The structure and properties of CPs can be controlled by adjusting the synthesis conditions [11]. These polymers have been utilized in a variety of technological applications, including electromagnetic shielding [160, 161, 162], antistatic coatings [163, 164, 165], rechargeable batteries [166, 167, 168], solar cells [169, 170, 171, 172], biomedical devices [173, 174, 175], smart textiles [176, 177], chemical and mechanical sensors [178, 179, 180, 181], and others. Additionally, they have been explored as extractor phases in sample preparation techniques, attributed to the unique characteristics of these materials, which can be enhanced through modifications in their synthesis and by the different interactions between the CPs (hydrogen bonding, π–π, electrostatic, and hydrophobic interactions) and the target compounds [2, 182]. Recent applications of CPs for the extraction of various organic compounds from environmental, food, and biological samples will be described in the following section.

#### 3.3.2 Recent Applications of CPs in Sample Preparation

##### 3.3.2.1 CPs in Environmental Matrices

CPs are playing an important role in advancing analytical approaches based on sample preparation techniques for monitoring complex environmental matrices such as soil and different types of water. Their use contributes to more efficient and accurate detection and quantification of target compounds.

In an interesting study conducted by Hajializadeh et al., the authors successfully developed a new fiber for the SPME composed of PANI, modified MWCNTs, and ZIFs. The applicability was proved to isolate and quantify trace amounts of some organic pollutants such as naphthalene, fluorene, phenanthrene, and anthracene in aqueous samples, both from environmental samples (well, sea, aqueduct, and tap water) and from food samples (different types of tea) [183]. The determination of these analytes is crucial due to their toxicity and to their potential mutagenic and carcinogenic effects. The main advantage of this method is its applicability to both environmental and food matrices. The analytes were quantified by GC‐flame ionization detector (FID), and the method achieved LOQs from 0.9 to 2.5 µg L^−1^ for both matrices. Recovery rates range between 83.4% and 111.2% for environmental samples and between 83.5% and 110.8% for food samples. Performing the process directly in headspace mode and without pH adjustment contributes to the green aspects of the method. Criterion 2 (Hazardous Materials) achieved the maximum score, indicating that the sample preparation does not involve the use of toxic substances. Generally, this is a differential of the SPME technique, where no or only minimal amounts of organic solvents are used. Consequently, an AGREEprep of 0.55 was obtained for this method, with waste generation limited to only 10 mL (sample volume). However, the sample preparation placement was ex situ (Criterion 1), and the sample throughput (Criterion 6) was not favorable.

Our research group has expertise in developing CP‐based extraction phases for application in sample preparation techniques. Huelsmann et al. [184] demonstrated the efficiency of PPy as an extraction phase in disposable pipette extraction (DPX) for the determination of 18 organic micropollutants in river and tap water samples. Given the versatility and chemical stability of PPy, the study aimed to evaluate its efficacy for extracting various classes of pollutants, including phthalic acid esters, personal care products, alkylphenols, organophosphorus pesticides, and organochlorine pesticides, which frequently contaminate aquatic systems. PPy was easily synthesized by the chemical oxidative synthesis, and it provided high extraction efficiency due to the possibility of several interactions between PPy and analytes. Its integration into DPX offered several advantages, such as simplified handling, reduced solvent consumption, and high sample throughput, which were later confirmed in the method's greenness assessment through AGREEprep. Following extraction, the samples were analyzed by GC–MS, which provided high sensitivity, with LOQs ranging from 1.4 to 5 µg L^−1^. The method proved to be an environmentally friendly (AGREEprep of 0.46) and viable approach for routine monitoring of water quality and assessing contaminants in these matrices. The key advantage observed was the possibility of preparing multiple samples simultaneously, which enhanced analytical throughput (Criterion 6), even though the procedure was performed manually, without the use of automated systems. However, some disadvantages should be noted, such as the use of GC–MS, which consumes more energy compared to other analytical instruments without MS detectors. Additionally, exposure to hazardous materials (Criterion 10) was higher compared to the other methods discussed here for the use of CPs in the analysis of environmental matrices.

In addition to PANI and PPy, PTh‐based materials have also been explored. A recent study by Heydari et al. [185] presented an innovative extraction phase named magnetic porous carbon covered with a polythiophene–polyindole (MPC@PTh‐PIn). MPC@PTh‐PIn was used in the dispersive micro‐solid‐phase extraction (D‐m‐MSPE) technique for the determination of phthalate esters from seawater, with subsequent analysis using GC‐FID. Comparative studies demonstrated that MPC@PPy‐PTh is more effective in extracting phthalates than either the base MOF or the intermediate MPC alone. The extraction conditions were optimized, yielding excellent analytical performance, with LOQs between 0.2 and 0.3 µg L^−1^, linear ranges from 0.2 to 100 µg L^−1^, and satisfactory recoveries (89%–104%). The study suggests that due to its unique features, MPC@PTh‐PIn could be useful for pre‐concentrating metal ions and anionic species in future environmental applications, providing an effective and sustainable tool for monitoring trace contaminants in complex matrices. The authors evaluated the green aspects of the method using the ComplexGAPI tool, a recent approach for assessing the environmental impact of analytical procedures. According to the authors, the highlights were that although the method's green rating is affected by the solvent consumption and high temperatures involved in the synthesis of MPC@PPy‐PTh, the method itself requires only 88 µL of ethyl acetate per sample. Additionally, it demonstrated relatively green characteristics based on the ComplexGAPI tool. Considering that the AGREEprep score was 0.27, this method may not be considered as green compared to other methods previously discussed. Despite using only 88 µL of solvent, which contributed positively to the score for Criterion 2, the sample size was relatively large (Criterion 5), specifically 96.5 mL, as was the waste generation (Criterion 4). In addition, the procedure involved multiple steps performed manually (Criterion 7).

Other applications of CPs in sample preparation for environmental samples are presented in Table 8 [183, 184, 185, 186, 187, 188, 189, 190, 191, 192, 193, 194, 195, 196, 197, 198, 199, 200, 201, 202, 203, 204, 205, 206].

**TABLE 8:** Recent applications of conductive polymers in sample preparation methods applying chromatographic separation for environmental samples.

Material	Sample preparation technique	Analytes	Sample	Instrument	Linear range	LOQ	Recovery (%)	AGREEprep	Reference
MWCNTs/ZIF‐67/PANI	SPME	Naphthalene, fluorene, phenanthrene, and anthracene	Well, sea, aqueduct, and tap water	GC‐FID	0.005–1000.0 µg L^−1^	0.9–2.5 µg L^−1^	83.4–111.2	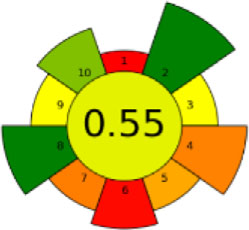	[183]
PPy	DPX	Dimethyl phthalate; diethyl phthalate; benzophenone; 4‐octylphenol; terbufos; 4‐nonylphenol; methyl parathion; dibutyl phthalate; metolachlor; chlorpyrifos; aldrin; 4‐methylbenzylidene camphor; 4,4′‐dichlorodiphenyldichloroethylene); endrin; 4,4′‐dichlorodiphenyldichloroethane; benzyl butyl phthalate; bis 2‐ethylhexyl phthalate, and di‐*N*‐octyl phthalate	River and tap water	GC–MS	1.4–100 µg L^−1^	1.4–5 µg L^−1^	74.9–116.2	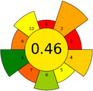	[184]
MPC@PTh‐PIn	D‐m‐MSPE	Di‐*N*‐propylphthalate, di‐*N*‐butyl phthalate, diethylhexyl adipate, and di(2‐ethylhexyl) phthalate	Seawater	GC‐FID	0.2–100 µg L^−1^	0.2–0.3 µg L^−1^	89–104	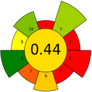	[185]
PANI‐etched MWCNT/UiO‐66‐NH_2_	SPME	Phenanthrene, fluoranthene, and pyrene	Lake water	HPLC–DAD	0.05–20 ng mL^−1^	0.03 ng mL^−1^	87.0–102.0	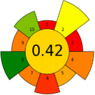	[186]
PANI/Pan NFsM	SPE	Aspirin, ketoprofen, naproxen, clofibric acid, diclofenac, indomethacin, ibuprofen, and tolfenamic acid	Sewage, wastewater, and drinking water	UHPLC–MS/MS	1.0 ng L^−1^–15.0 µg L^−1^	0.6–15.0 ng L^−1^	85.0–99.7	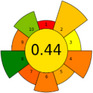	[187]
PANI	SPE	Fluorene, naphthalene, acenaphthylene, anthracene, acenaphthene, benz[A]anthracene, and pyrene	Tap water, well water, river water, and wastewater	GC‐FID	0.01–100 ng mL^−1^	—	95.8–99.7	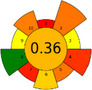	[188]
Fe_3_O_4_@SiO_2_@PANI	DSPE	2‐Chorophenol, 4‐chlorohenol, 2,4‐dichlorophenil, and 2,4,6‐trichlorophenol	Tap water, industrial wastewater, and river water	HPLC‐UV	3–100 µg L^−1^	1.07–2.00 µg L^−1^	85.13–98.54	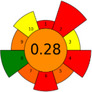	[189]
SiO_2_/PANI	NTD	Naphthalene, acenaphthene, fluorene, anthracene, phenanthrene, fluoranthene, pyrene, benzene, toluene, ethylbenzene, and xylene	Soil	GC‐FID	0.2–2000 ng g^−1^	—	92.36–115.21	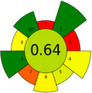	[190]
Sulfonated PANI NFM	SPE	Norfloxacin, ciprofloxacin, ofloxacin, enrofloxacin, danofloxacin, pefloxacin, marbofloxacin, lomefloxacin, and difloxacin	Lake, river, and tap water	UHPLC–MS/MS	1.5–50000 ng L^−1^	1.5–5.0 ng L^−1^	83.7–109.0	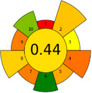	[191]
PANI/TiO_2_ nanorods	SPME	Dimethyl phthalate, diethyl phthalate, diallyl phthalate, benzyl butyl phthalate, di‐*N*‐butyl phthalate, di‐*N*‐pentyl phthalate, and dicyclohexyl phthalate	Bottled water	HPLC–DAD	0.03–30 µg L^−1^	—	81.0–115.2	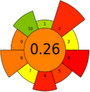	[192]
PANI‐PDMS	SBSE	Estradiol, ethinylestradiol, estrone, diethyl stilbestrol, and hexestrol	Lake and river water	HPLC‐UV	0.5–500 µg L^−1^	—	86.6–106	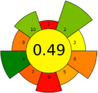	[193]
PANI	SPME	Dimethyl methylphosphonate, trimethyl phosphate, triethyl phosphate, 1,4‐thioxane, 2‐chloroethyl ethyl sulfide, diphenylchlorarsine, and phenyldichloroarsine	Soil	GC–MS/MS	0.02–5000 ng g^−1^	0.02–0.45 ng g^−1^	96.2–101.2	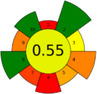	[194]
Fe_3_O_4_@GO‐PANI	MSPE	Fluorene, phenanthrene, pyrene, 2‐nitrofluorene, 9‐nitroanthracene, 1‐nitropyrene, and 3‐nitrofluoranthene	Mineral, tap, and river water	GC–MS	0.2–75 ng mL^−1^	0.04–0.33 ng mL^−1^	91.6–108.8	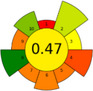	[195]
GO@LDH@SPANI	UA‐d‐SPE	Dimethyl phthalate, dibutyl phthalate, benzyl butyl phthalate, di‐(2‐ethylhexyl) phthalate, and benzyl benzoate	Drinking water	GC–MS	0.2–1000 ng mL^−1^	0.2–1 ng mL^−1^	92.3–107.7	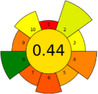	[196]
PPy‐Au‐BaCP	SPME	3‐Nitrobiphenyl, 9‐nitroguanidine, 9‐nitrophenanthrene, 3‐nitrofluoranthene, and 1‐nitroguanidine	Lake water, river water, and drinking water	GC‐FID	10–3500 ng L^−1^	—	80.3–118.0	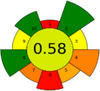	[197]
PPy	SPE	Ametryn, atrazine, promethrin, propazine, simazine, simetryn, and terbutryn	River water	HPLC‐UV	0.5–50 ng mL^−1^	0.5 ng mL^−1^	74.8–105.0	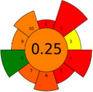	[198]
PPy/TiO_2_	SPME	2‐Chlorophenol, 2,4‐dichlorophenol, 2,6‐dichlorophenol, and triclosan	River, wastewater, and tap water	HPLC‐UV	0.05–200 µg L^−1^	0.03–0.09 µg L^−1^	89.7–112	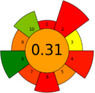	[199]
PPy	SPE	Atrazine, caffeine, and progesterone	River water	GC–MS	5.0–500 µg L^−1^	5–50 µg L^−1^	83.2–89.6	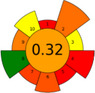	[200]
OPAC‐Fe_3_O_4_‐PPy	MSPE	Endosulfan and dieldrin	Tap and river water, palm Oil mill effluent	GC‐ECD	25–1000 ng L^−1^	22–25 ng L^−1^	95.58–103.5	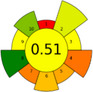	[201]
PPy	SPME	Diazinon and chlorpyrifos	Well water	GC–MS	0.25–300.0 ng mL^−1^	0.07–0.1 ng mL^−1^	88.5–104.1	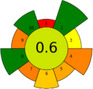	[202]
MPC@PPy‐PTh	DµSPE	4‐Chlorophenol 2‐naphtol, 1‐amino‐2‐naphthol, 2,4‐dichloroaniline, 3,4‐dichloroaniline, benzothiophene, and naphthalene	Seawater and wastewater	HPLC–DAD	0.25–500 µg L^−1^	0.25–0.5 µg L^−1^	85–106	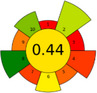	[203]
PPy.CTAB	DPX	17α‐Ethynylestradiol, estrone, acenaphthylene, fluorene, anthracene, phenanthren, pyrene, chrysene, benzo[a]anthracene, benzo[b]fluoranthene, benzo[*k*]fluoranthene, benzo[a]pyrene, dibenz[a,h]anthracene, and benzo[g,h,i]perylene	River water	HPLC–DAD	5–125 µg L^−1^	5–20 µg L^−1^	44–139	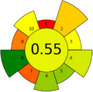	[204]
MWCNT/MnO_2_/PEDOT	SPME	Naphthalene, 1‐methyl naphthalene, acenaphthene, and fluorene	Soil	GC‐FID	0.5–250 ng g^−1^	—	87–108	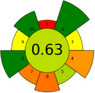	[205]
HP‐PEDOT/PVA	IS‐SPE	Methyl paraben, ethyl paraben, propyl paraben, and butyl paraben (parabens)	Mineral water	HPLC–DAD	0.5–100 µg L^−1^	0.5–1 µg L^−1^	88.5–96.1	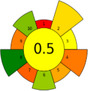	[206]

Abbreviations: Fe_3_O_4_@GO‐PANI, magnetic graphene oxide nanocomposite modified with polyaniline; Fe_3_O_4_@SiO_2_@PANI, polyaniline functionalized silica coated magnetic nanoparticles; HP‐PEDOT/PVA, hierarchically porous fabricated using polyethylene dioxythiophene embedded in a polyvinyl alcohol cryogel; IS‐SPE, in‐syringe‐solid‐phase extraction; MPC@PPy‐PTh, polypyrrole‐polythiophene coated magnetic porous carbon; MWCNT/MnO_2_/PEDOT, multi‐walled carbon nanotubes/manganese dioxide nanocomposite‐based polythiophene; MWCNTs/ZIF‐67/PANI, polyaniline modified multi‐walled carbon nanotubes and zeolitic imidazolate frameworks; NTD, needle trap device; OPAC‐Fe_3_O_4_‐PPy, oil‐palm fiber activated carbon modified with magnetite and polypyrrole; PANI NFM, polyaniline nanofiber mat; PANI/Pan NFsM, polyaniline/polyacrylonitrile nanofibers mat; PANI/TiO_2_, polyaniline/titanium dioxide; PANI‐etched MWCNT/UiO‐66‐NH_2_, polyaniline composite doped with etched multi‐walled carbon nanotubes and UiO‐66‐NH_2_; PANI‐PDMS, polyaniline‐polydimethylsiloxane; PPy.CTAB, polypyrrole.cetyltrimethylammonium bromide; PPy‐Au‐BaCP, stable gold nanoparticles and barium coordination polymer doped polypyrrole; SiO_2_/PANI, polyaniline/silica.

The AGREEprep obtained for the methods reported in Table 8 ranged from 0.25 to 0.64. A critical assessment of the 24 studies revealed that most methods achieved metrics below 0.5, whereas only 9 exceeded this value. Methods with higher metrics (>0.5) were those that minimized the consumption of hazardous materials, consequently reducing waste generation, and employed sustainable materials that were renewable and/or reusable. Conversely, methods that required larger sample quantities and involved multiple sample preparation steps achieved metrics between 0.25 and 0.49. All the methods employed chromatography (liquid or gas) as the instrumental technique, which involves high energy consumption and negatively impacts the greenness of the methods.

##### 3.3.2.2 CPs in Food Samples

CPs and their applicability in sample preparation techniques have gained significant attention in the search to determine various compounds in food samples. They are highly effective in the extraction and detection of contaminants due to their selectivity and ease of integration into SPE‐based techniques.

A notable example of using CPs was demonstrated by Otoukesh et al. [196], who employed a GO/LDHs@sulfonated PANI (SPANI) nanocomposite for ultrasonic‐assisted dispersive solid‐phase extraction (UA‐d‐SPE) of phthalates, including dimethyl phthalate, dibutyl phthalate, benzyl butyl phthalate, di‐(2‐ethylhexyl) phthalate, and benzyl benzoate in various beverages. Phthalates are commonly used as plasticizers in food packaging and have been identified as potential endocrine disruptors, making their detection in food matrices crucial for ensuring consumer safety. The validated method was applied to distilled herbal beverages such as mint, rose, and chicory, as well as drinking water. The LOQs ranged from 0.2 to 1 ng mL^−1^, with recovery rates between 54.5% and 112.6% for distilled herbal beverages and 92.3%–107.7% for drinking water. Although a recovery rate of 54.5% was obtained, as suggested by the authors, the method showed good repeatability and accuracy, demonstrating its effectiveness across different matrices. Moreover, the AGREEprep for this method was calculated, and a score of 0.44 was obtained. Some advantages and disadvantages are presented, such as lower use of hazardous materials, the possibility of reusability of GO@LDH@SPANI, and low energy consumption, which do not negatively impact the score for Criteria 2, 3, and 8, respectively. Considering that semi‐automated or fully automated systems were not used, the sample preparation is done manually, involving some steps, and this limits the number of samples processed per hour, thereby reducing the sample throughput.

García‐Nicolás et al. explored the use of MDSPE as an innovative sample preparation technique for the determination of aflatoxins in paprika [207]. Aflatoxins are highly toxic secondary metabolites produced by certain fungi, and their presence in food products poses serious health risks, including carcinogenic effects, making their detection and control critical for food safety. This method employed a magnetic nanocomposite coated with PPy in MDSPE to selectively extract aflatoxins B1, B2, G1, and G2 from paprika. The MDSPE technique was optimized to ensure high extraction efficiency, minimal solvent consumption, and reduced sample preparation time. This method was successfully validated, with LOQs ranging from 3.5 to 4.7 µg kg^−1^. Additionally, the method provided excellent recovery rates, typically ranging between 81.9% and 99.4%, and demonstrated high repeatability and reproducibility under various experimental conditions. The application of MDSPE to paprika samples revealed its potential not only for routine monitoring of aflatoxins in spices but also for broader applications in food safety. This method offers a simple, cost‐effective, and eco‐friendly alternative, with an AGREEprep of 0.43, similar to the previously discussed study (AGREEprep of 0.44) [196]. Advantages include the small sample size required (Criterion 5), needing only 0.2 g of paprika, and lower waste generation (Criterion 4), both of which contribute to the method's greenness.

**FIGURE 3:**
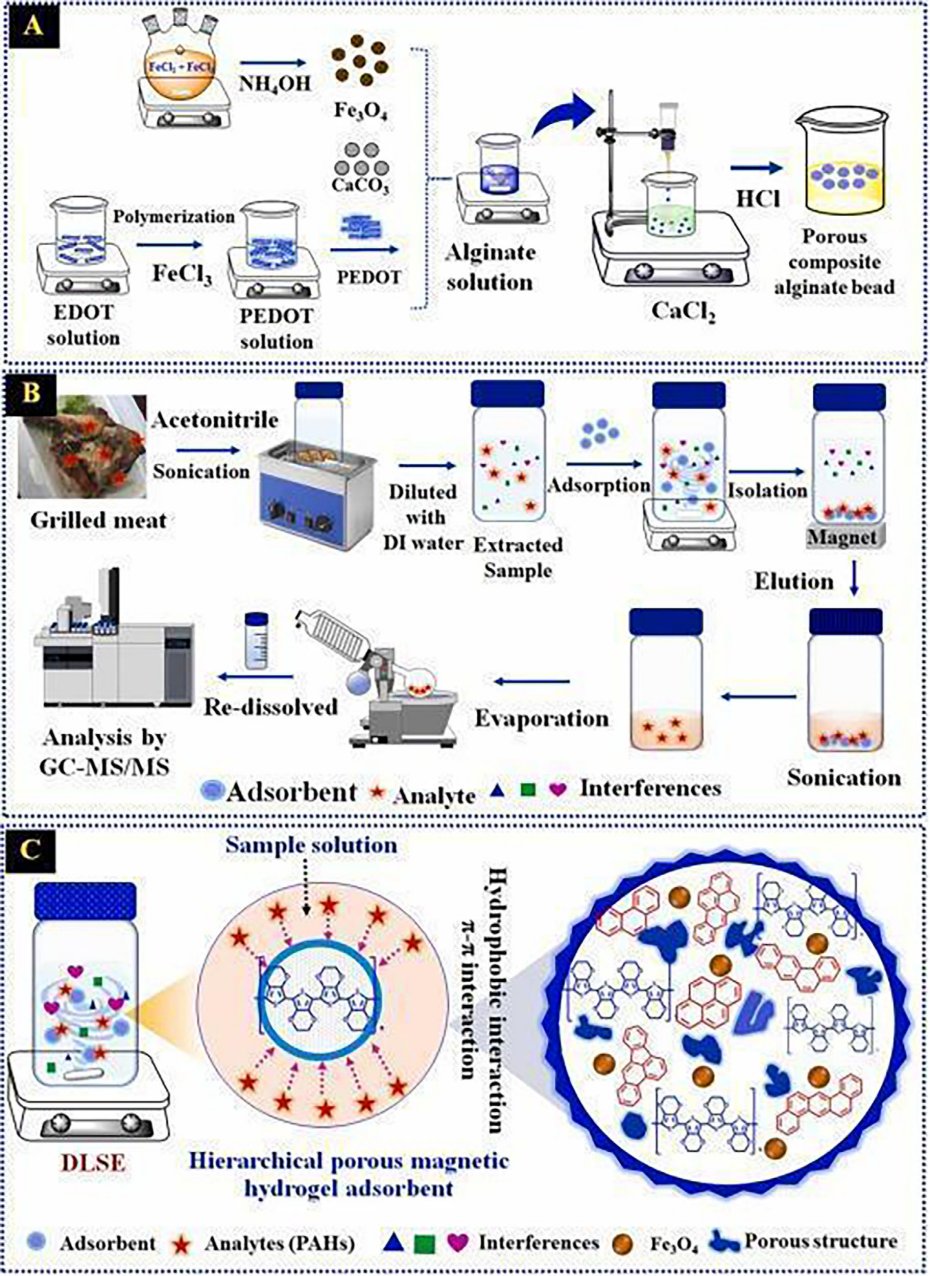
The schematic illustrations of synthesis procedure of hierarchically porous material fabricated using magnetic alginate hydrogel beads impregnated with PEDOT (A), the DLSE procedure of polycyclic aromatic hydrocarbons using the hierarchically porous material fabricated using magnetic alginate hydrogel beads impregnated with PEDOT (B), and the interaction between the analytes and hierarchically porous material fabricated using magnetic alginate hydrogel beads impregnated with PEDOT. DLSE, dispersive liquid–solid‐phase extraction; PEDOT, poly(3,4‐ethylenedioxythiophene). *Source*: Jullakanet al. [208] with permission from Elsevier, copyright 2022.

Another study conducted by Jullakan et al. involved a hierarchically porous material fabricated using magnetic alginate hydrogel beads impregnated with poly(3,4‐ethylenedioxythiophene) (PEDOT@Fe_3_O_4_@AL) for dispersive liquid–solid‐phase extraction (DLSE) of five polycyclic aromatic hydrocarbons (PAHs), including pyrene, benz[*a*]anthracene, benzo[*b*]fluoranthene, benzo[*a*]pyrene, and dibenz[*a*,*h*]anthracene [208]. The adsorbent material with a hierarchical nanoporous structure was synthesized (Figure 3A) and applied in DLSE technique for the analysis of PAHs, which are widely regulated due to their toxicity and carcinogenic potential. The interaction between PAHs and PEDOT@Fe_3_O_4_@AL is illustrated in Figure 3C. The extraction process was optimized using minimal solvents, emphasizing the reduced solvent consumption as an environmentally friendly alternative compared to traditional methods. Furthermore, the authors achieved good validation results, demonstrating that the developed DLSE coupled with the GC–MS/MS method is efficient, showing good accuracy in three different meat samples, with satisfactory recoveries (81.5%–99.4%). Compared to the other studies discussed that involve the use of CPs in food sample analysis, this work involved higher waste generation (Criterion 4), required more sample preparation steps (Criterion 7) as shown in Figure 3B, and had greater hazards associated with the procedure (Criterion 10). Both methods discussed in this section received the same scores for Criterion 1 (sample preparation placement), Criterion 3 (sustainability, renewability, and reusability of materials), and Criterion 9 (post‐sample preparation configuration for analysis).

Other applications of CPs in sample preparation for food samples are presented in Table 9 [183, 193, 196, 202, 206–218].

**TABLE 9:** Recent applications of conductive polymers in sample preparation methods applying chromatographic separation for food samples.

Material	Sample preparation technique	Analytes	Sample	Instrument	Linear range	LOQ	Recovery (%)	AGREEprep	Reference
MWCNTs/ZIF‐67/PANI	SPME	Naphthalene, fluorene, phenanthrene, and anthracene	Green, black, chamomile, and borage tea infusion	GC‐FID	0.005–1000 µg L^−1^	0.9–2.5 µg L^−1^	83.5–110.8	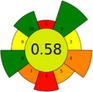	[183]
PANI‐PDMS	SBSE	Estradiol, ethinylestradiol, estrone, diethyl stilbestrol, and hexestrol	Chicken and pork	HPLC‐UV	0.5–500 µg L^−1^	—	82.0–111	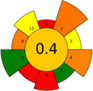	[193]
GO@LDH@SPANI	UA‐d‐SPE	Dimethyl Phthalate, dibutyl phthalate, benzyl butyl phthalate, di‐(2‐ethylhexyl) phthalate, and benzyl benzoate	Distilled herbal beverages	GC–MS	0.2–1000 ng mL^−1^	0.2–1 ng mL^−1^	54.5–112.6	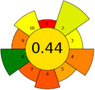	[196]
PPy	SPME	Diazinon and chlorpyrifos	Commercial apple and grape juices, and natural apple, and tomato juices	GC–MS	0.25–300.0 ng mL^−1^	0.07–0.1 ng mL^−1^	93.2–107.6	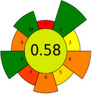	[202]
HP‐PEDOT/PVA	IS‐SPE	Methyl paraben, ethyl paraben, propyl paraben, and butyl paraben	Flavored water, juice, beer, and milk	HPLC–DAD	0.5–100 µg L^−1^	0.5–1 µg L^−1^	88.4–98.4	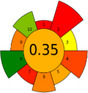	[206]
Fe_3_O_4_@PPy	MDSPE	Aflatoxin G1, G2, B1, and B2	Paprika	UPLC‐HRMS	3.5–50 µg kg^−1^	3.5–4.7 µg kg^−1^	81.9–99.4	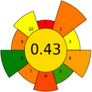	[207]
HP‐PEDOT@Fe_3_O_4_@AL	DLSE	Pyrene, benz[a]anthracene, benzo[b]fluoranthene, benzo[a]pyrene, and dibenz[a,h]anthracene	Grilled fish, grilled chicken, and grilled pork	GC–MS/MS	0.50–200.0 µg kg^−1^	0.5–1.0 µg kg^−1^	81.5–99.4	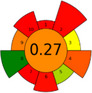	[208]
MWCNTs/PANI‐PPy@PDMS	SPME	Hexachlorobenzene, chlorothalonil, fipronil, and chlorfenapyr	Garlic	GC–MS	1–400 ng g^−1^	1.27–6.33 ng g^−1^	84.0–108.2	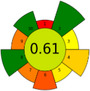	[209]
emerald‐based PANI‐PAN NFMs	SPE	Sudan Dyes (Sudan I–IV)	Poultry feed	HPLC–DAD	25–10 000 µg kg^−1^	25–50 µg kg^−1^	88.48–101.88	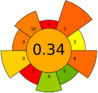	[210]
PANI‐PAN NFMs	SPE	Paracetamol and chloramphenicol	Meat	UHPLC–MS/MS	0.03–200 µg kg^−1^	0.03–0.7 µg kg^−1^	87.6–108.3	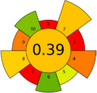	[211]
PANI/GO	SPME	Oxytetracycline, tetracycline, and doxycycline	Bovine milk	HPLC‐UV	8.05–750 µg L^−1^	8.05–25.27 µg L^−1^	71–104	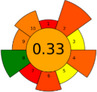	[212]
PPy/PDA	SPME	Abscisic acid, gibberellic acid, and indole acetic acid	Fruit juices	HPLC‐UV	0.02–20 µg mL^−1^	0.02–0.05 µg mL^−1^	89.6–110.0	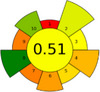	[213]
PA/GO/PPy	SC‐µSPE	Methyl paraben, ethyl paraben, and propyl paraben	Milk	HPLC‐UV	10–1000 ng mL^−1^	10–20 ng mL^−1^	81.7–97.8	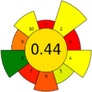	[214]
PPy‐carbon	SPME	Pyrimethanil, cyprodinil, kresoxim‐methyl, and trifloxystrobin	Grape juice	GC–MS	0.6–50 ng mL^−1^	0.55–6.94 ng mL^−1^	81.4–99.8	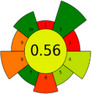	[215]
CFs@PPy	SPME	2‐Pentylfuran	Coffee	GC–MS	0.1–50 ng mL^−1^	—	84–102	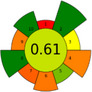	[216]
Cu@PPy@HNTs@Fe_3_O_4_	MSPE	Sulfathiazole, sulfamerazine, sulfamonomethoxine, and sulfadimethoxine	Milk	HPLC–DAD	2.5–150.0 µg kg^−1^	7.5–10.0 µg kg^−1^	83.0–99.2	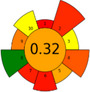	[217]
PTh	PP and MEPS	Progesterone, prednisolone, and estradiol	Bovine milk	HPLC–DAD	16–1200 ng mL^−1^	16 ng mL^−1^	88.29–98.68	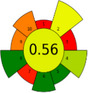	[218]

Abbreviations: CFs@PPy, carbon fibers with polypyrrole; Cu@PPy@HNTs@Fe_3_O_4_, metallic copper coated on polypyrrole polymer composited with halloysite nanotubes decorated with magnetite nanoparticles; Fe_3_O_4_@PPy, magnetic nanocomposite coated with polypyrrole; HP‐PEDOT/PVA, hierarchically porous fabricated using polyethylene dioxythiophene embedded in a polyvinyl alcohol cryogel; MWCNTs/PANI‐PPy@PDMS, multiwalled carbon nanotubes/polyaniline‐polypyrrole@polydimethylsiloxane; PA/GO/PPy, polyamide‐graphene oxide‐polypyrrole; PANI/GO, polyaniline/graphene oxide; PANI‐PAN NFMs, polyaniline‐modified polyacrylonitrile nanofiber mats; PP, protein precipitation; PPy/PDA, pyrrole‐dopamine copolymers.; PPy‐carbon, polypyrrole modified with carbonaceous nanomaterials.

The greenness of the methods presented in Table 9 was evaluated using AGREEprep. In these 17 studies, the metrics ranged from 0.27 to 0.61. Among them, most did not achieve metrics that could be considered “green” (>0.5). This can be attributed to the fact that most of the techniques employed were not miniaturized, resulting in higher sample consumption, waste generation, and often greater exposure to hazardous materials. Additionally, the lack of automated systems and the multiple steps involved in sample preparation negatively impacted the metrics of these methods. Future advancements should be proposed to improve the sustainability of methods and, thus, highlight their potential.

##### 3.3.2.3 CPs in Biological Samples

CPs have also shown great potential in sample preparation techniques for isolating and concentrating analytes in complex biological matrices such as blood, urine, and serum. The use of CPs in biological sample preparation helps to simplify the extraction process, reduce solvent consumption, and increase the extraction recovery.

In a remarkable study by Ghaedrahmati et al., the authors developed an SPME method by combining MOF with PANI for the extraction and detection of endogenous aldehydes in biological fluids such as plasma and urine samples [219]. Aldehydes such as heptanal and hexanal are important biomarkers in the human body, and their presence in biological fluids is suitable for investigations due to their elevated levels in biological fluids, particularly in patients with lung cancer. The use of this nanocomposite sorbent in an SPME method offers several advantages. For example, it presents higher mechanical and chemical resistance and a longer lifetime. The inclusion of PANI, a CP, in a magnetic MOF not only boosts the adsorption capacity but also provides enhanced selectivity. Although the authors did not report the LOQs, recoveries were obtained in the range of 92.5%–122.1%, which can be considered adequate given the complexity of these samples. This study achieved the AGREEprep of 0.58. This score reflects the environmentally friendly nature of the SPME in headspace mode technique, which eliminates the need for organic solvents, making the method more environmentally friendly and significantly enhancing operator safety under Criterion 10, “Operator's safety,” with “No hazards or no exposure.” Waste generation (Criterion 4) was limited to only 10 mL, corresponding to the sample volume used, further supporting the greenness of the method. The sample throughput (Criterion 6) was favorable, with up to five samples processed per hour. Although the sample preparation placement (Criterion 1) was “ex situ,” this method effectively balances efficiency and sustainability.

In 2023, our research group published a study that explored the use of a novel extraction phase based on PANI‐silica doped with oxalic acid, which was applied in the TF‐SPME technique for the determination of hormones in urine [220]. Hormones such as estrogen steroids are critical biomarkers, and their accurate detection is essential for clinical diagnostics and health studies. These compounds often exist at trace levels in complex biological matrices, necessitating effective sample preparation techniques to achieve reliable results. PANI‐silica doped with oxalic acid was synthesized through aniline oxidation. Ammonium persulfate was used as the oxidant, whereas oxalic acid served as the dopant, promoting the final conductivity and adsorption properties. Validation results demonstrated satisfactory results, such as LOQs from 1 to 10 µg L^−1^ and relative recoveries ranging from 71% to 115%. This methodology employed a novel extraction phase aimed at improving extraction efficiency while reducing solvent use and sample preparation time, using a 96‐well plate sampling system. This study achieved an AGREEprep of 0.42, which is lower than that of the previously discussed study [219] (AGREEprep of 0.58). For Criteria 1 (sample preparation placement) and 3 (sustainability, renewability, and reusability of materials), both methods achieved the same score, whereas differences were found in the other criteria. The scores for hazardous materials (Criterion 2), energy consumption (Criterion 8), post‐sample preparation configuration for analysis (Criterion 9), and operator's safety (Criterion 10) were negatively impacted. Because this method involved greater use of hazardous materials and increased energy consumption, as well as the use of HPLC‐FLD rather than GC‐FID, these factors contributed to a lower score in sustainability and safety. However, this method has advantages, including lower waste generation (Criterion 4) and size economy of the sample (Criterion 5), requiring only 1.5 mL of sample, as well as high sample throughput (Criterion 6) with a semi‐automated system (Criterion 7), whereas the other method used a manual system.

In addition to PANI, PPy has also been explored in sample preparation techniques, as demonstrated by Zanganeh et al. [221]. In their study, they developed a composite material made from a COF and PPy to act as the sorbent in an MEPS for the efficient extraction of opiates such as codeine, papaverine, and naltrexone from urine samples [221]. The use of HPLC‐UV as a detection method in combination with the MEPS technique is very relevant for the determination of opiates, which are commonly found in biological matrices due to the use of these drugs. The authors mentioned that the combination of COF and PPy provided several advantages, including high specific surface area, chemical and thermal stability, and an increase in the number of active sites for efficiently extracting the opioids through acid‐base and π–π interactions. The study highlights that the proposed method offers low LOQs, good reproducibility, and high recovery rates (94.4%–103.1%), suggesting its applicability for routine analysis in clinical and forensic toxicology. Additionally, the use of MEPS offers a greener alternative to traditional extraction, as reflected in its AGREEprep of 0.45, placing it among the scores obtained for the articles discussed involving the use of CPs for biological sample analysis [219, 220]. Both methods used “ex situ” sample preparation placement (Criterion 1). Compared to other methods, this approach has some disadvantages. The use of hazardous materials (Criterion 2) was relatively high, sample throughput (Criterion 6) was lower, and more sample preparation steps were required, along with the use of “manual systems” (Criterion 7), which negatively affected the scores for these criteria. Waste generation (Criterion 4) was intermediate, whereas sample size economy (Criterion 5) was reduced.

Other applications of CPs in sample preparation for biological samples are shown in Table 10 [191, 198, 219–226].

**TABLE 10:** Recent applications of conductive polymers in sample preparation methods applying chromatographic separation for biological samples.

Material	Sample preparation technique	Analytes	Sample	Instrument	Linear Range	LOQ	Recovery (%)	AGREEprep	Reference
Sulfonated PANI NFM	SPE	Norfloxacin, ciprofloxacin, ofloxacin, enrofloxacin, danofloxacin, pefloxacin, marbofloxacin, lomefloxacin, and difloxacin	Urine and serum	UPLC–MS/MS	0.04–1000 µg L^−1^	0.04–0.17 µg L^−1^	83.9–109.6	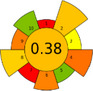	[191]
PPy	DPX	Dexamethasone	Synthetic synovial liquid	HPLC‐UV	10–100 ng mL^−1^	10 ng mL^−1^	89.5–94.3	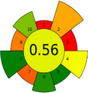	[198]
Fe_3_O_4_@MIL‐101(Cr)/PANI	SPME	Hexanal and heptanal	Plasma and urine	GC‐FID	0.1–1 µg L^−1^	—	95.2–122.1	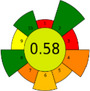	[219]
PANI‐silica doped with oxalic acid	TF‐SPME	17β‐Estradiol, 17α‐ethinylestradiol, and estrone	Urine	HPLC‐FLD	1–500 µg L^−1^	1.0–10.0 µg L^−1^	71–115	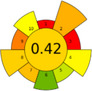	[220]
COF‐PPy‐CTAB	MEPS	Codeine, papaverine, and naltrexone	Urine	HPLC‐UV	0.5–1000 µg L^−1^	0.5–5 µg L^−1^	94.4–103.1	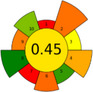	[221]
PANI‐NFs	SPME	Tamoxifen	Urine	GC‐FID	2–1130 µg L^−1^	1.7 µg L^−1^	89–106	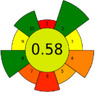	[222]
PCL‐PANI	EC‐SPME	Losartan, irbesartan, and valsartan	Blood plasma	HPLC‐UV	5–2000 µg L^−1^	3–6.1 µg L^−1^	91.1–104.3	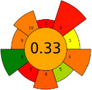	[223]
NGPPC	DSPE	Methamphetamine	Urine	HPLC‐UV	30–800 ng mL^−1^	29 ng mL^−1^	99.76	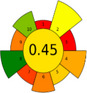	[224]
Fe_3_O_4_@PPy NPs	MSPE	Glibenclamide	Urine and serum	HPLC‐UV	0.2 –700.0 µg L^−1^	0.350 µg L^−1^	92.0–102.5	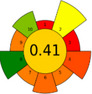	[225]
Fe_3_O_4_@PPy	MSPE	Folic acid and riboflavin	Urine	UPLC‐DAD	0.23–12.5 µg mL^−1^	0.07–0.18 µg mL^−1^	92.2–105.1	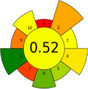	[226]

Abbreviations: COF‐PPy‐CTAB, covalent organic framework‐polypyrrole‐cetyltrimethylammonium bromide; EC‐SPME, electrochemically controlled‐solid‐phase microextraction; Fe_3_O_4_@MIL‐101(Cr)/PANI, magnetic metal–organic framework/polyaniline nanocomposite; Fe_3_O_4_@PPy NPs, polypyrrole‐modified magnetic nanoparticles; NGPPC, nano graphene oxide polypyrrole composite; PANI‐NFs, polyaniline nanofibers; PANI‐silica doped with oxalic acid, polyaniline‐silica doped with oxalic acid; PCL‐PANI, polyaniline immobilized on polycaprolactam nanofibers.

Through the evaluation of the greenness of the methods presented in Table 10, most of the methods developed using CPs in sample preparation techniques for the analysis of biological samples exhibited metrics below 0.5; specifically, this was 6 out of the 10 reported studies, with values ranging from 0.33 to 0.58. These methods employed ex situ sample preparation placement, which contributed to the lower metrics. Furthermore, high waste generation was observed in some cases, with up to 20 mL of waste produced. According to the reported data and the considerations made for the metric calculation, most of the methods did not employ automated or semi‐automated systems, and in many cases, multiple steps were involved in the sample preparation process.

## 4 Conclusions and Prospects

Recent advances in materials science have significantly impacted the field of sample preparation techniques, with materials such as MIPs, MOFs, and CPs emerging as pivotal players in the development of green analytical methodologies. These materials exhibit remarkable properties, including high selectivity, tailored porosity, and enhanced stability, making them ideal for addressing the challenges associated with traditional sample preparation methods. In terms of future prospects, there are different directions for each of the materials involved.

A tendency in the use of MIPs is that advances will be achieved in the synthesis stage, with the aid of computational studies, in order to improve efficiency and reproducibility, allowing the prediction and optimization of interactions between the polymer and the analyte, the use of fictitious MIP models applied to a class of analytes as well as adaptations to the principles of green chemistry. Furthermore, there will likely be an expansion of MIP applications, such as electrochemical sensors, cell recognition, and application as quality control in food samples, due to the combination of MIPs with nanomaterials that have improved properties.

MOFs continue to be developed due to their versatile compositions and synthesis methods. However, challenges remain regarding their use in different sample matrices, particularly in environments with varying pH levels, elevated temperatures, or solvents that may cause decomposition or degradation. Their application in biological matrices is still relatively unexplored, representing a promising avenue for future research. Regarding their synthesis, some MOFs already incorporate DES and other biopolymers, but further exploration of these green materials could enhance sustainability and improve their compatibility with environmentally friendly sample preparation techniques.

CPs have also demonstrated significant potential in sample preparation. Future research could focus on designing CP‐based materials with enhanced selectivity through molecular imprinting strategies or functionalized composites. Additionally, improving CPs’ stability under varying pH conditions and organic solvent exposure is crucial for broadening their applicability. Finally, the combination of CPs with emerging nanomaterials, such as COFs or MOFs, may lead to hybrid materials with synergistic properties, further improving extraction performance.

The integration of sustainable practices—such as reduced solvent consumption and environmentally friendly synthetic routes—has enabled these materials to align increasingly with the principles of green chemistry. However, some limitations remain, especially when methods are evaluated using tools such as AGREEprep. Another critical issue is the low adoption of multivariate optimization methods, as most studies rely on univariate approaches and do not adequately explore the interactions between variables during the method development stage.

Future prospects in the field are notably encouraging. Key research directions include the development of multifunctional hybrid materials, the miniaturization and automation of analytical platforms—such as microfluidic systems and lab‐on‐a‐chip devices—enhanced biocompatibility, and the incorporation of renewable precursors. The integration of biobased monomers and recyclable feedstocks, combined with life‐cycle assessment approaches, is expected to reduce the environmental impact of analytical methodologies. The convergence of innovative material engineering and green chemistry principles is anticipated to further advance sample preparation techniques. As these materials become more established, their application is likely to expand across diverse areas, including environmental analysis, food safety, and biomedical diagnostics, promoting more sustainable and high‐performance analytical practices.

## Author Contributions

**Eduardo Carasek**: conceptualization, resources, supervision, project administration, investigation, funding acquisition, writing – review and editing. **Amanda Vitória Santos**: investigation, writing – original draft, conceptualization. **Francielle Crocetta Turazzi**: conceptualization, investigation, writing – original draft. **Lucas Morés**: conceptualization, investigation, writing – original draft. **Luciane Effting**: conceptualization, investigation, writing – original draft. **Guilherme Mariz de Oliveira Barra**: conceptualization, writing – original draft, supervision.

## Data Availability

The authors have nothing to report.
